# Characterization of TGF-β by Induced Oxidative Stress in Human Trabecular Meshwork Cells

**DOI:** 10.3390/antiox10010107

**Published:** 2021-01-13

**Authors:** Hsin-Yi Chen, Hsiu-Chuan Chou, Yi-Jung Ho, Shing-Jyh Chang, En-Chi Liao, Yu-Shan Wei, Meng-Wei Lin, Yi-Shiuan Wang, Yu-An Chien, Xin-Ru Yu, Hsiang-Yu Kung, Chu-Chun Yang, Jia-Yu Chen, Hong-Lin Chan, Mei-Lan Ko

**Affiliations:** 1 Institute of Bioinformatics and Structural Biology & Department of Medical Sciences, National Tsing Hua University, Hsinchu 300, Taiwan; dful690@gmail.com (H.-Y.C.); nakyla1215@gmail.com (E.-C.L.); t91050127@hotmail.com.tw (Y.-S.W.); eva1018cat@gapp.nthu.edu.tw (M.-W.L.); woowoow0320@gmail.com (Y.-S.W.); eileenyaeileen@gmail.com (Y.-A.C.); yang19980314@gmail.com (C.-C.Y.); 2 Department of Biomedical Engineering and Environmental Sciences, National Tsing Hua University, Hsinchu 300, Taiwan; chouhc@mail.nhcue.edu.tw (H.-C.C.); kelly.s9220030@gmail.com (X.-R.Y.); a86145259a@gmail.com (H.-Y.K.); jiayu2811@gmail.com (J.-Y.C.); 3 Department of Ophthalmology, National Taiwan University Hospital Hsin-Chu Branch, Hsinchu 300, Taiwan; phify66@gmail.com; 4 Department of Obstetrics and Gynecology, Hsinchu MacKay Memorial Hospital, Hsinchu 300, Taiwan; Justine3@ms8.hinet.net

**Keywords:** TGF-β signal pathway, oxidative stress, trabecular meshwork cells, fibrosis

## Abstract

Oxidative stress generated by reactive oxygen species (ROS) plays a critical role in the pathomechanism of glaucoma, which is a multifactorial blinding disease that may cause irreversible damage within human trabecular meshwork cells (HTMCs). It is known that the transforming growth factor-β (TGF-β) signaling pathway is an important component of oxidative stress-induced damage related to extracellular matrix (ECM) fibrosis and activates cell antioxidative mechanisms. To elucidate the dual potential roles and regulatory mechanisms of TGF-β in effects on HTMCs, we established an in vitro oxidative model using hydrogen peroxide (H_2_O_2_) and further focused on TGF-β-related oxidative stress pathways and the related signal transduction. Via a series of cell functional qualitative analyses to detect related protein level alterations and cell fibrosis status, we illustrated the role of TGF-β1 and TGF-β2 in oxidative stress-induced injury by shTGF-β1 and shTGF-β2 knockdown or added recombinant human TGF-β1 protein (rhTGF-β1). The results of protein level showed that p38 MAPK, TGF-β, and its related SMAD family were activated after H_2_O_2_ stimulation. Cell functional assays showed that HTMCs with H_2_O_2_ exposure duration had a more irregular actin architecture compared to normal TM cells. Data with rhTGF-β1 (1 ng/mL) pretreatment reduced the cell apoptosis rate and amount of reactive oxygen species (ROS), while it also enhanced survival. Furthermore, TGF-β1 and TGF-β2 in terms of antioxidant signaling were related to the activation of collagen I and laminin, which are fibrosis-response proteins. Succinctly, our study demonstrated that low concentrations of TGF-β1 (1 ng/mL) preserves HTMCs from free radical-mediated injury by p-p38 MAPK level and p-AKT signaling balance, presenting a signaling transduction mechanism of TGF-β1 in HTMC oxidative stress-related therapies.

## 1. Introduction

Glaucoma is a progressive and irreversible optic neuropathy, and one of the dominant causes of blindness worldwide [1]. Elevated intraocular pressure (IOP) is a major risk factor leading to the development of visual field defects associated with the disease [2]. The IOP is controlled by the balance between the secretion and drainage of aqueous humor. In humans, aqueous humor is drained primarily by human trabecular meshwork cells (HTMCs), which are situated in an iridocorneal angle. IOP elevation is believed to be based on dysfunction or increased resistance of the HTMCs to the outflow of aqueous humor [3]. Owing to the cytopathological changes caused by the expression and activity of oxidative stress factors [4,5], normal dysfunction of HTMCs can lead to an increase in IOP. It has been reported that the trabecular meshwork has high levels of lipid peroxidation metabolites and a large number of DNA adducts [6,7]. Oxidation and biomolecular damage to HTMCs have been considered the cause of the obstruction of aqueous humor outflow. Furthermore, in pulmonary fibrosis, epilepsy, hypertension, atherosclerosis, Parkinson′s disease, and sudden death, oxidative stress plays an important role and is also believed to be associated with many ophthalmic disorders, such as age-related macular degeneration, cataracts, and glaucoma [8]. In several ocular cells, including corneal epithelial cells (CECs), HTMCs, retinal pigment epithelial cells (RPEs), and retinal ganglion cells (RGCs), oxidative stress may occur. In particular, oxidative stress-induced dysfunction of HTMCs can suppress the outflow of aqueous humor, lead to pathologically high IOP, and lead to glaucoma [9]. As a result, the progression of glaucoma has been linked to oxidative stress, which can result from decreased expression and activity of antioxidant proteins. Several publications have appeared in recent years documenting that increased deposits of extracellular matrix (ECM) in HTMCs are responsible for elevated IOP [10,11]. Senescence markers, such as cyclin-dependent kinase (CDK) inhibitors, p16 and p21, are activated by elevated cellular oxidative load. Induction of p16 and p21 is specifically correlated with ROS growth accumulation in aging and glaucomatous HTMCs. All these results suggest that oxidative stress and senescence/aging are closely related. Moreover, recent research based on cell culture supports the deleterious role of oxidative stress in HTMC abnormalities [12].

HTMC is in regular contact with aqueous humor that has been demonstrated to have reactive oxygen species (ROS) and modify trabecular meshwork physiological function. ROS is also responsible for cellular signaling, including transforming growth factor-βs (TGF-βs) with various cytokines and growth factors [13]. It has been shown that TGF-βs are present in the ocular media of humans and other species, and the content of TGF-β increases in aqueous humor during aging/glaucoma [1,14,15]. In addition, TGF-β-induced ECM protein overstimulation has been shown in the trabecular meshwork of glaucoma/aging, and this process is thought to be involved in the pathobiology of HTMCs [16]. TGF-βs are activated by ROS during oxidative stress. It is worth noting that they are also inducers of ROS [17,18], which act by regulating the Nicotinamide adenine dinucleotide phosphate (NADPH) oxidase 4 (NOX4) enzyme [19]. Excess ROS can result in cell damages by over-stimulation of genes such as ECM protein genes by synergistic signaling caused by the TGF-β and ROS processes.

TGF-β is a multifunctional growth factor that is associated with many biological processes, such as cell growth, differentiation, cell survival, adhesion, apoptosis, ECM production, and neuroprotection [1]. TGF-β activates downstream signal transduction after binding to its receptors (serine/threonine kinases, TGF-βR), such as mitogen-activated protein kinase (MAPK) signaling pathway activation, and SMAD2/3 phosphorylation, which is a potential concomitant reaction of oxidative-related pathways. Despite the undetermined etiology and pathophysiology of glaucoma, many studies have shown that the ECM of the HTMCs, oxidative stress, TGF-β signaling pathways, and apoptosis are critically linked to glaucoma pathogenesis [3,20,21,22]. These leading factors seem to be correlated, but mechanistic analysis with respect to correlation remains incomplete [22]. TGF-βs have also been shown to directly cause increased IOP [23]. In the anterior chamber of glaucomatous patients, substantial upregulation of TGF-β content has been evaluated and induces changes in protein level expression, including various heat-shock proteins, components of the ubiquitin proteasome pathway, antioxidants, and DNA repair-related enzymes, while several proteins involved in mitochondrial oxidative phosphorylation show downregulation in the ocular hype [24]. One of the notable phenomena for adjacent cell damage during ischemia is the elevation of Ca^2+^ concentration. Intracellular metabolism can be interfered with by increased concentrations of Ca^2+^ and apoptosis through several pathways. The free radicals produced in this process can further intensify or damage fibrosis [25]. In addition, the signals of TGF-β may be transmitted through collateral signaling pathways, such as the pathways of Akt/PI3K, p38 MAP kinase, ERK, and Rho-associated kinase (ROCK) [26]. Previous studies show that, in HTMCs, the ROCK pathway is involved in regulating resistance in the outflow of aqueous humor and IOP [27,28]. A broad family of proteins triggered by a wide variety of proinflammatory cytokines and environmental stressors are included in MAPKs. In cellular processes, MAPKs play key roles in proliferation, apoptosis, control of the genes, differentiation, and motility [29,30].

H_2_O_2_ cell stimulation is a widely used method for induction of oxidative stress damage in vitro, especially in order to create an efficacy of antioxidant and pathway recognition in oxidatively damaged HTMCs [31]. Although H_2_O_2_ treatment is a general procedure to produce stress stimulation in cell culture, it is difficult to regulate optimized concentration in the cultured medium during cell exposure. As such, the concentration and treating exposure period of H_2_O_2_ have been optimized in this study to imitate the oxidative status in cell microenvironments.

In HTMCs, TGF-β can also promote cytoskeletal remodeling, cell adhesion, and ECM production. In this study, we analyzed the role of TGF-β signaling in HTMCs. Mechanistic analyses of HTMCs showed that oxidative stress-activated p-p38 signaling and TGF-β1 with their associated pathway concomitantly increased in terms of activation, leading to ECM deposition and further causing HTMC dysfunction, while the dual potential roles in TGF-β1 showed protective effects under specific conditions.

## 2. Materials and Methods

### 2.1. Reagents and Antibodies

Lipofectamine^®^ RNAiMAX transfection reagent was purchased from Invitrogen (Thermo Fisher Scientific Inc., Waltham, MA, USA) and OPTI- minimal essential medium (MEM) was purchased from Gibco (Thermo Fisher Scientific Inc., Waltham, MA, USA). MTT (3-(4,5-dimethylthiazol-2-yl)-2,5-diphenyltetrazoliumbromide) was purchased from USB Corp. (Thermo Fisher Scientific Inc., Waltham, MA, USA). Annexin-V/propidium iodide (PI) Conjugate Detection kit was purchased from Life Technologies Corp. (Carlsbad, USA). Anti-rabbit and anti-mouse immunoglobulin (Ig) G horseradish peroxidase (HRP)-conjugated secondary antibodies were purchased from Jackson ImmunoResearch Laboratories, Inc. (Baltimore, PA, USA). Anti-rabbit and anti-mouse immunoglobulin (Ig)G fluorescein isothiocyanate (FITC)-conjugated secondary antibodies were purchased from SeraCare KPL (Washington, Maryland, USA). Phospho-JNK, JNK, PI3K, phospho-AKT, AKT, vimentin, phospho-SMAD2, phospho-SMAD3, SMAD2/3, SMAD4, SMAD7, and lactate dehydrogenase (LDH) primary antibodies were purchased from Genetex Inc. (Hsinchu, Taiwan). Phospho-p38 MAPK (Thr180/Tyr182) and p38 MAPK primary antibodies were purchased from Cell Signaling Technology (Danvers, MA, USA). Calreticulin, calmodulin, TGF-β1, TGF-β2, alpha-smooth muscle actin (α-SMA), and collagen I primary antibodies were purchased from ABclonal (Woburn, MA, USA). All chemicals and reagents used in this study were of analytical grade.

### 2.2. Cell Line and Cell Culture

HTMCs, which were isolated from the juxtacanalicular and corneoscleral regions of the human eye, were purchased from ScienCell Research Laboratories (Carlsbad, USA, Catalog #6590). Cells were cultured in trabecular meshwork cell medium (TMCM) supplemented with fetal bovine serum (FBS), trabecular meshwork cell growth supplement, and penicillin/streptomycin solution at 37 °C in a 5% CO_2_ incubator.

### 2.3. H_2_O_2_ Sufficiency

Hydrogen peroxide (30%) was purchased from Sigma-Aldrich (Thermo Fisher Scientific Inc., Waltham, MA, USA). Before the treatment, we first prepared the solution with a final concentration of 20 mM in serum-free medium. The reagent was diluted to specific concentration in serum-free medium before addition to the cells. After the duration time, we stopped the H_2_O_2_ treatment with twice 1× phosphate buffered saline (PBS) washing, and further followed by ROS reagent staining, apoptosis assay, Western blot sample preparing, or other functional assays.

### 2.4. shTGF-β1/2 Knockdown HTMC Establishment

The lentivirus package plasmid (pMD.G, pCMVDR8.91), control plasmid (pLKO.1), and anti-TGF-β1 and shTGF-β2 shRNA constructs were purchased from RNAiCore (Academia Sinica, Taiwan). HEK-293T cells were transfected with pLKO1, shTGF-β1/2 constructs, pMD.G, and PCMVDR8.91 plasmids using X-tremeGENE transfection reagent (Roche Diagnostics) to generate shTGF-β1/2 lentivirus when cells achieved 70% convergence. The transfection reagent X-tremeGENE and plasmids were diluted with the medium OPTI-MEM (Invitrogen, Thermo Fisher Scientific Inc.). After 24 h of transfection, the medium was refreshed, and the supernatant containing lentivirus particles was collected after 72 h and filtered through 0.45 μm filters. HTMCs were transduced with pLKO.1, shTGF-β1, and shTGF-β2 lentivirus particles in 2 mL of complete medium containing polybrene (8 μg/mL) and incubated at 37 °C and 5% CO_2_ for 48 h. The transduced HTMCs underwent antibiotic selection in medium containing 0.3 μg/mL puromycin twice to become shTGF-β1 and shTGF-β2 stable knockdown cell lines.

### 2.5. siRNA Knockdown and Transfection

The small interfering RNA (siRNA) was designed and synthesized by Invitrogen against p38 MAPK (Waltham, USA). The targeting sequence against p38 MAPK was 5′–CCU AAA ACC UAG UAA UCU ATT–3′. Cell seeding was at a density of 3 × 10^5^ cell/6 cm dish for 24 h of incubation, and transfection was mediated using the Lipofectamine^®^ RNAiMAX Transfection Reagent according to the manufacturer′s instructions. In brief, the cells were transfected through an OPTI-MEM medium containing Lipofectamine^®^ for 4 h with 40 nM p38 MAPK siRNA (sip38 MAPK) or a corresponding negative control (siCtrl; GE Healthcare Dharmacon Inc., Lafayette, USA). The cells were then recovered for at least 24 h in new and complete medium. The same procedure was repeated for the secondary siRNA knockdown. The efficiency of siRNA knockdown was usually within five days and monitored by immunoblotting analysis.

### 2.6. Cell Viability Assay

Viability of cells was detected with the MTT solution (USB Corp., Cleveland, OH, USA). Cells at a density of 8 × 10^3^ cells per well were seeded into 96-well plates. The media were discarded after 24 h of incubation, and the cells were stimulated with various concentrations and H_2_O_2_ exposure durations for the specified time courses. The H_2_O_2_-containing medium was removed and the cells were further incubated for 3.5 h at 37 °C in the dark in 100 µL of MTT solution (1 mg/mL). The MTT solution was then extracted, and 100 μL of dimethyl sulfoxide (DMSO) per well was added. To completely dissolve the insoluble purple formazan, the 96-well plates were shaken for 30 s, and the absorbance of each well was measured at 570 nm by a plate reader.

### 2.7. Detection of ROS Increase

ROS increase was measured using aminophenyl fluorescein (APF; Goryo Chemical, Sapporo, Japan). To prepare the solution with a final concentration of 5 mM, 1 mg of APF powder was dissolved in 0.47 mL of *N*,*N*-dimethylformamide (DMF). Before being applied to the cells, the reagent was diluted to 5 μM using phosphate-buffered saline (PBS) or serum-free medium. Following the addition of APF, the cells were incubated for 15 min in the dark and the fluorescence emission at 490 nm was measured at 515 nm after excitation. Accuri CFlow@ and CFlow Plus tools were used to measure the extent of APF fluorescence (BD Biosciences) [32]. The fluorescence shift percentage was normalized to that of the control group.

### 2.8. Intracellular Calcium-Level Measurement

The intracellular free calcium calculation was performed with Fluo-8^®^ dye (AAT Bioquest, Sunnyvale, USA). To prepare the solution with a final concentration of 5 mM, a total of 0.25 mg of Fluo-8^®^ dye powder was dissolved into 0.48 mL of DMSO. Before being applied to the cells, the reagent was diluted to 5 μM using PBS or serum-free medium. The cells were incubated in the dark for 20 min after the addition of Fluo-8^®^ solution, and the fluorescence emission was measured at 520 nm post excitation at 490 nm. Accuri CFlow@ and CFlow Plus tools were used to measure the extent of Fluo-8^®^ fluorescence (BD Biosciences). The percentage of the fluorescence change was normalized to that of the control group.

### 2.9. Apoptosis Assay

A total of 1 × 10^6^ cells were collected and resuspended in 1 mL of binding buffer following H_2_O_2_ stimulation. Then, 5 μL of Alexa Fluor 488-conjugated annexin V and PI (5 μL 100 μg/mL) were used to stain cells (100 μL) as directed by the manufacturer. Then, the samples were gently mixed and incubated in the dark for 15 min at 4 °C. Samples were filtered after staining and subjected to a BD Accuri C6 flow cytometer (BD Biosciences, San Jose, CA, USA). The results were analyzed using CFlow Plus tools software (BD Biosciences) [33].

### 2.10. Immunoblotting Analysis

The quantified protein samples (60 µg/μL) were extracted and separated on a 12% gel and transferred to polyvinylidene difluoride (PVDF) membranes (Pall Corp., Port Washington, WI, USA). The primary antibodies (Genetex Inc., Hsinchu, Taiwan) were diluted 1:2000 after membranes were blocked with 5% (*w*/*v*) skimmed milk or bovine serum albumin (BSA) in Tris-buffered saline with Tween-20 (TBST; 50 mM Tris, 150 mM NaCl, and 0.1% Tween-20 (*v*/*v*); pH 8.0) for 1 h, and applied to the membranes that were incubated overnight at 48 h at 4 °C. Thus, the membranes were washed six times (10 min/wash) in TBST, and then incubated with a 1:10,000 dilution of correct HRP coupling secondary antibody (Jackson ImmunoResearch Laboratories, Inc.) solutions for 1 h with gentle restlessness. The membranes were then washed six times (10 min/time) again in TBST, and the protein analysis was visualized using a system of enhanced chemiluminescence (ECL) (Visual Protein Biotech Corp., Taipei, Taiwan). Image J software was used to analyze the data from the densitometric quantification on the basis of the ratio to housekeeping protein (lactate dehydrogenase; LDH). A two-dimensional Western blot to separate the different protein species before identifying the proteins and the post-translational modifications (PTM) might be even more efficient. For JNK, with the same antibody, sometimes one band and sometimes two bands are seen [34,35]. We verified the same trend of the band in every condition of the independent experiments, and we analyzed the double bands, with one being the native form of the protein, and the other one being the PTM form. Quantification of both represented the total protein expression.

### 2.11. Immunofluorescence

The cells were seeded onto 12 mm coverslips (VWR International Corp., Radnor, USA) plated with a density of 4 × 10^4^ cells per well in 24-well plates and incubated for adhesion overnight. The cells were then fixed at 37 °C for 25 min with PBS containing 4% (*v*/*v*) paraformaldehyde (PFA) and washed with 1× PBS three times. The coverslips were incubated in PBS containing 0.1% (*v*/*v*) Triton X-100 for 5 min to permeabilize the cell membrane. Covers were washed and blocked in PBS containing 5% (*w*/*v*) BSA for 1 h before incubation with phalloidin tagged with selectively binding F-actin fluorescent dyes diluted in 2.5% BSA/PBS for optimized time at a ratio of 1:40. Coverslips were then washed with 1× PBS three times, sealed with glass slides with 4′,6-diamidino-2-phenylindole (DAPI) containing ProlongTM Antifade Mounting Medium (Thermo Fisher Science, Waltham, USA), and then deposited in the dark overnight at 4 °C. Samples were imaged using a Zeiss Axiovert 200 M fluorescent microscope for study (Carl Zeiss, Oberkochen, Germany). Images were exported with Zeiss Axioversion 4.0 as. zvi files and edited with Adobe Photoshop, v7.0 software.

### 2.12. Treatment of Cells with Recombinant Human TGF-β Protein1 (rhTGF-β1)

The cells were placed in a serum-free medium for 24 h before being exposed to the 5 ng/mL rhTGF-β1 protein for 24 h or left untreated in the control group. Whole-cell extracts were prepared for further immunoblotting analysis, ROS detection, or MTT assay in the NP40 cell lysis buffer.

### 2.13. Statistical Analysis

Data and figures are plotted as mean ± standard error of the mean (SEM). Differences between the experimental groups were assessed using paired Student’s *t*-test or one-way/two-way analysis of variance (ANOVA). Test results with *p* < 0.05 were considered statistically significant (* *p* < 0.05, ** *p* < 0.01, and *** *p* < 0.001).

## 3. Results

### 3.1. Impairments by H_2_O_2_ of HTMCs

**Figure 1:**
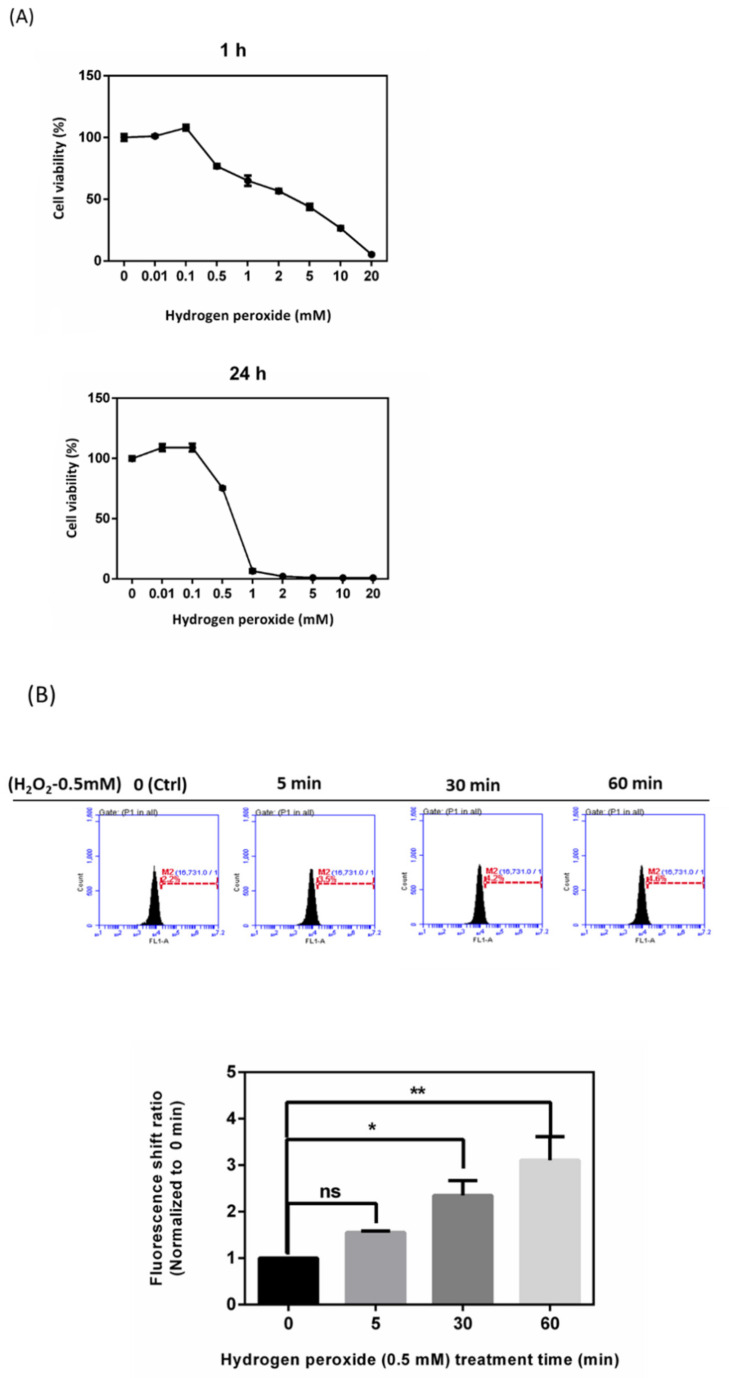

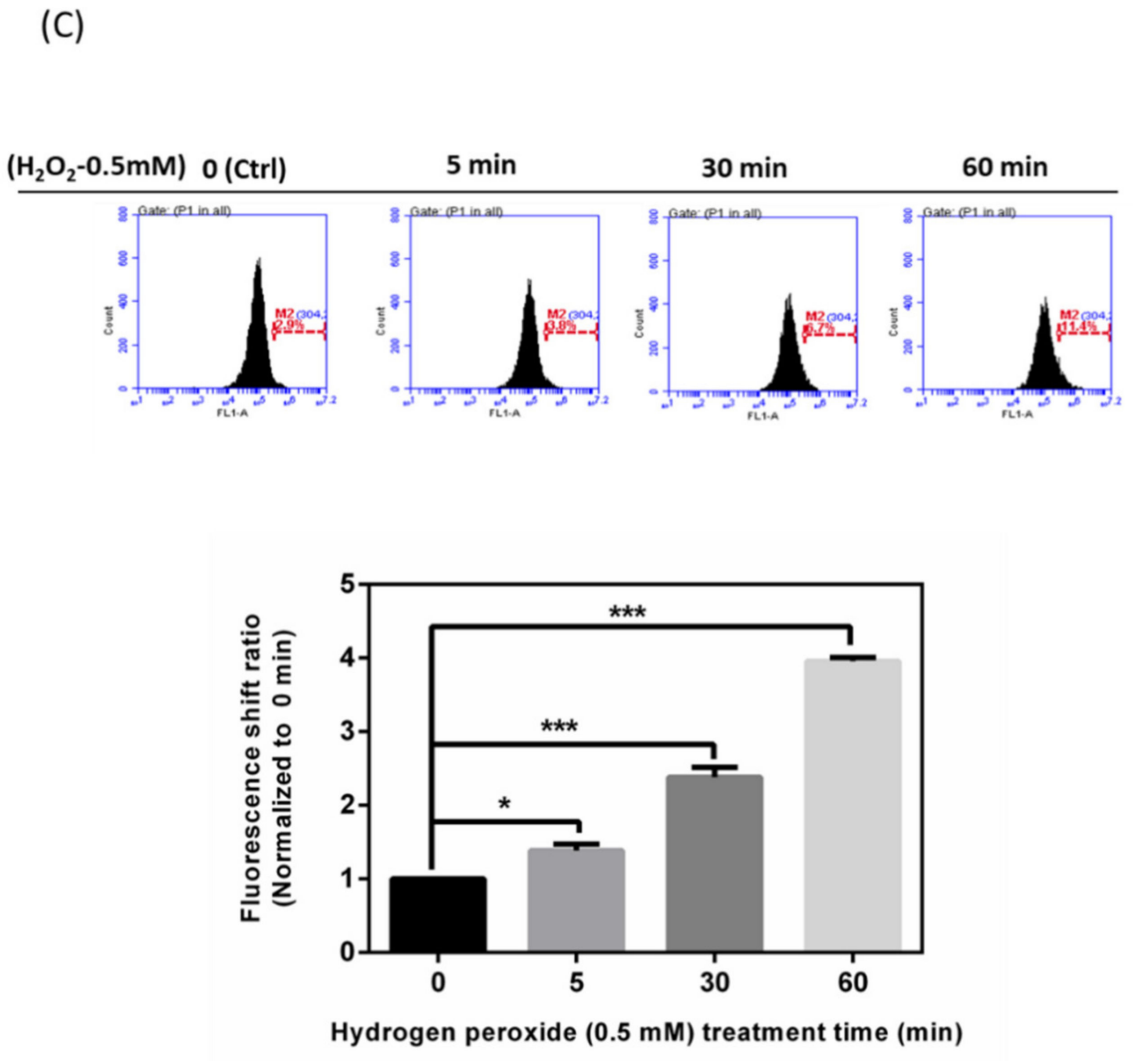
Impairments by H_2_O_2_ of human trabecular meshwork cells (HTMCs). H_2_O_2_ treatment decreased cell viability and increased reactive oxygen species (ROS) along with Ca^2+^ levels in dose- and time-dependent manners. (**A**) MTT (3-(4,5-dimethylthiazol-2-yl)-2,5-diphenyltetrazoliumbromide) cell viability of different concentrations of H_2_O_2_ for 1 (upper panel) and 24 h (lower panel) of treatment was evaluated. The various concentrations were normalized to 0 µM. Histogram values reflect the mean ± standard error of the mean (SEM). Values in the plots are normalized against the control group (0 mM) (*n* = 4, independent experiments). (**B**) The increase in ROS with H_2_O_2_ (0.5 mM)-treated HTMC over 5, 30, and 60 min. Histogram values reflect the mean ± SEM. Values in the plots are normalized against the control group (0 min); * *p* < 0.05, ** *p* < 0.01, and ns, non-significant when compared to the control group (*n* = 3, independent experiments). (**C**) Histograms showing decreased levels of Ca^2+^ content during H_2_O_2_ (0.5 mM) treatment. Values reflect the mean ± SEM and are normalized against the control group (0 min); * *p* < 0.05 and *** *p* < 0.001 when compared to the control group (*n* = 3, independent experiments).

Cell viability examination revealed that HTMC viability with H_2_O_2_ treatment decreased in a dose-dependent manner, and treatment with 0.5 mM H_2_O_2_ for 1 h decreased cell viability to 78.9% ± 1.2%; cell viability was reduced to less than 50% for 24 h (Figure 1A). To avoid H_2_O_2_ decay during exposure, we chose the duration time of 1 h and an exposure concentration of 0.5 mM H_2_O_2_ to be our optimized concentration in subsequent experiments. To explore time-dependent intracellular ROS after treatment with 0.5 mM H_2_O_2_, APF staining and flow cytometry were applied to analyze the ROS increase by altered signal percentages compared to the peak of the control group. ROS were detected at an exposure time of 5 min to 60 min, which increased the fluorescence shift ratio from 1.6 to 3.13 (Figure 1B). The cytosolic Ca^2+^ ([Ca^2+^]i) was estimated using Fluo-8^®^ dyes, which are specific Ca^2+^-binding fluorescent probes. Data showed that calcium accumulation increased fourfold in 60 min when compared to 0 min in the ocular cells under oxidative stress induced by H_2_O_2_ (Figure 1C).

### 3.2. Immunoblot Analysis and Identification of H_2_O_2_-Induced Oxidative Stress Response Signaling Pathways

**Figure 2:**
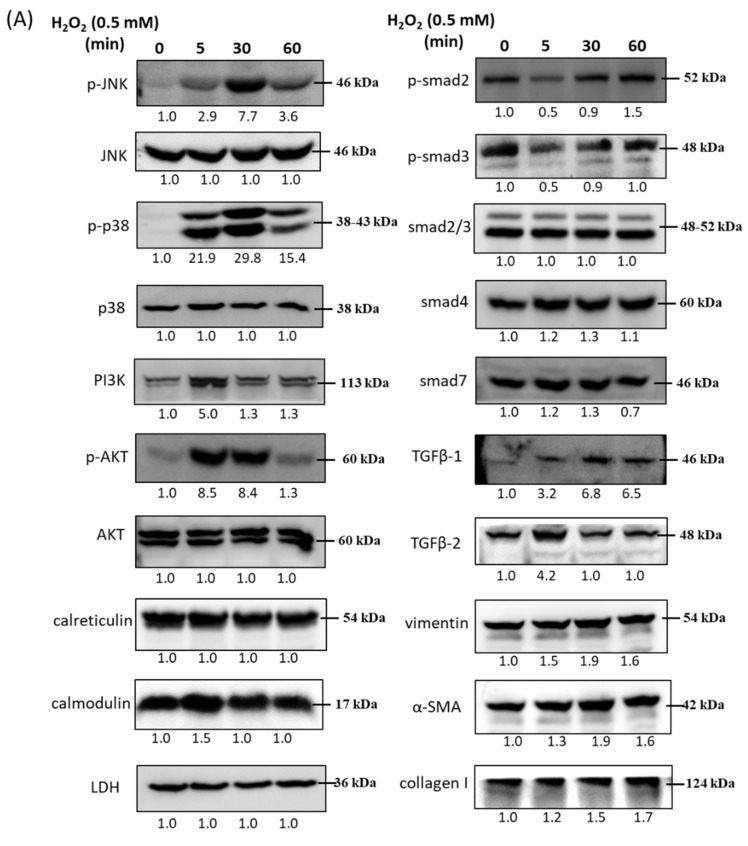

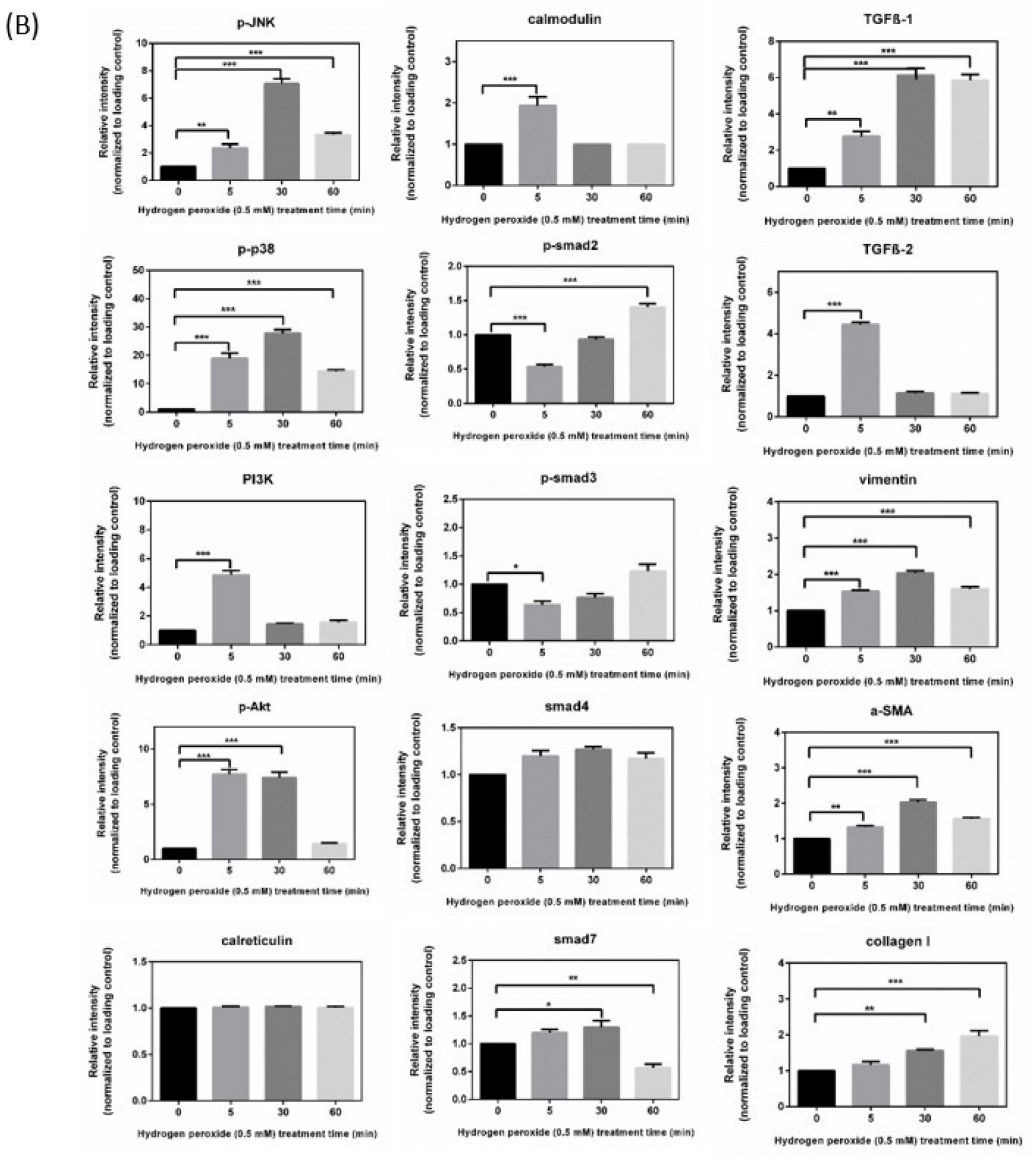
Immunoblot analysis and identification of H_2_O_2_-induced oxidative stress response signaling pathways. (**A**) Cells were collected after treatment with specific H_2_O_2_ concentration (0.5 mM) for 5, 30, and 60 min, and cellular extracts were immunoblotted with p-JNK, JNK, p-p38, p38, PI3K, p-AKT, AKT, calreticulin, calmodulin, p-SMAD2, p-SMAD3, SMAD2/3, SMAD4, SMAD7, transforming growth factor (TGF)β-1, TGFβ-2, vimentin, α-SMA, and collagen I, as shown with protein bands. Oxidative stress-dependent increases in ECM proteins, stress response, and TGF-β family members were observed over time. Numbers under each protein band reflect the densitometry value. (**B**) The protein expression intensities were quantified in relation to the loading control. Statistic chart were represented as means ± SEM. *, *p* < 0.05; **, *p* < 0.01; ***, *p* < 0.001 (*n* = 3).

HTMCs were treated with 0.5 mM H_2_O_2_ and the related signaling molecules were examined by immunoblotting 5, 30, and 60 min after beginning H_2_O_2_ treatment. Compared with the control, the protein levels of p-JNK, p-p38, and p-Akt were significantly increased at 30 min after treatment with 0.5 mM H_2_O_2_ and subsequently declined, while PI3K level increased within 5 min. The fold of calcium-related proteins, calreticulin and calmodulin, did not significantly change after treatment with 0.5 mM H_2_O_2_. In addition, the level of p-SMAD2 and p-SMAD3 decreased in 5 min but recovered up to 60 min. SMAD2/3 and SMAD4 did not significantly change, while SMAD7 showed a slightly decreased level at 60 min. We also examined the level of TGF-β1, TGF-β2, and the ECM markers, vimentin, α-SMA, and collagen I. The level of TGF-β1 increased continuously during 60 min of H_2_O_2_ treatment; thus, TGF-β2 level increased in 5 min and declined to that in the control. The protein level of vimentin, collagen I, and α-SMA slightly increased at 30 to 60 min (Figure 2).

### 3.3. Immunofluorescence Analysis of H_2_O_2_ (0.5 mM)-Treated HTMCs

**Figure 3:**
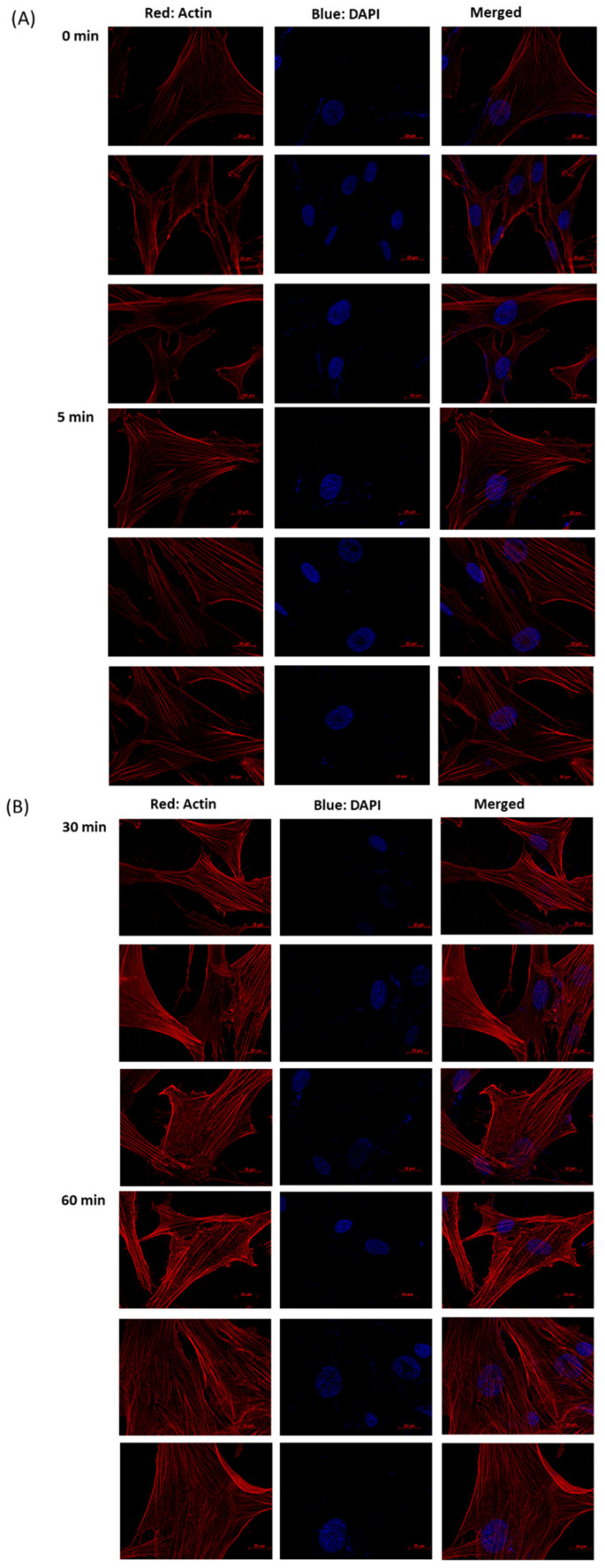

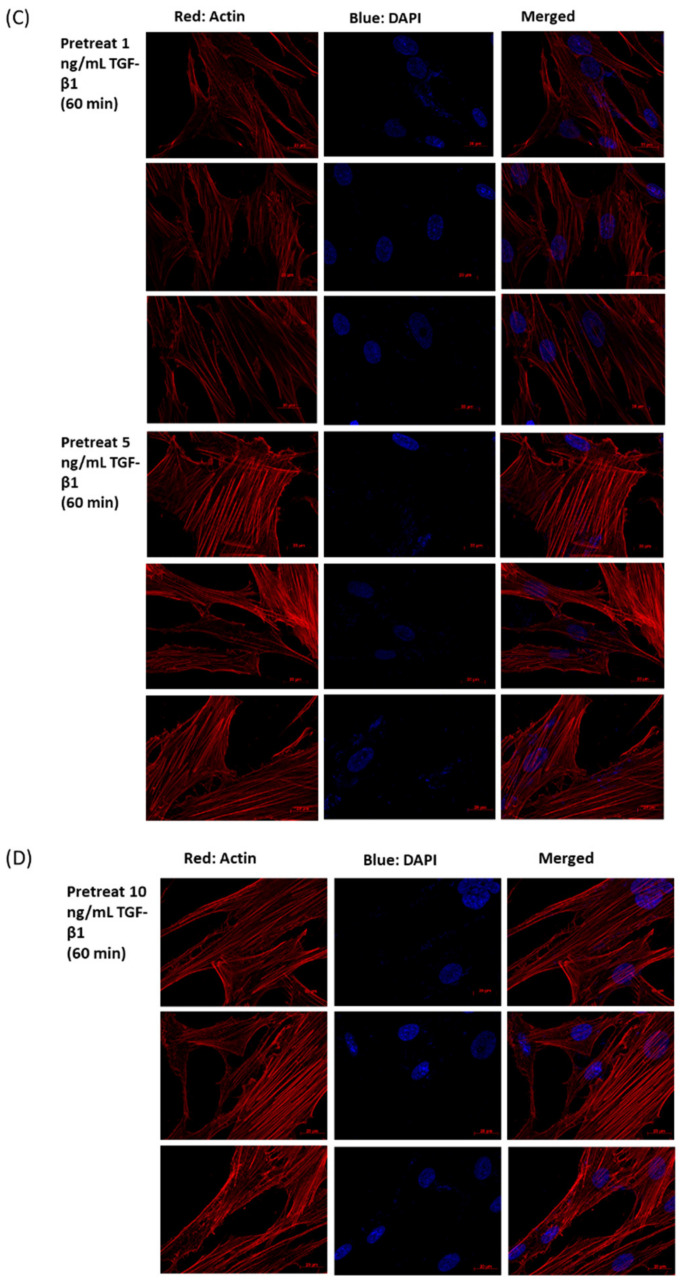
Immunofluorescence analysis of H_2_O_2_ (1 mM)-treated HTMCs. Photomicrograph of HTMCs with H_2_O_2_-treatment for (**B**) 30 and 60 min expressing more abnormal stress fiber formation, i.e., much denser and thicker compared to the (**A**) control and at a 5 min duration. HTMCs were treated with H_2_O_2_ on 12 mm coverslips for various exposure times (5, 30, 60 min) and were fixed and stained with phalloidin and 4′,6-diamidino-2-phenylindole (DAPI). (**C**,**D**) HTMCs pretreated with rhTGF-β1 in different concentrations (1, 5, 10 ng/mL) were observed for the prevention of fibrosis. The same exposure was used for each set of fields and the images are reflective of three different fields (scale bar = 20 µm).

Immunofluorescence staining revealed that the TM cells with longer H_2_O_2_ exposure duration (30 to 60 min with 0.5 mM H_2_O_2_) have a more irregular actin architecture and distribution, which presented thicker and depolymerized stress fibers, compared to normal TM cells. In addition, HTMCs pretreated with rhTGF-β1 in different concentrations (1, 5, 10 ng/mL) were observed for the prevention of fibrosis. Data showed that rhTGF-β1 (1 ng/mL) reduced irregular actin formation. These results suggest that morphological changes in HTMCs were accompanied by cytoskeletal rearrangement and led to cell fibrosis during oxidative stress, and rhTGF-β1 (1 ng/mL) treatment could prevent fibrosis (Figure 3).

### 3.4. Effects of rhTGF-β1 Protein on Oxidative Stress Response in HTMCs

**Figure 4:**
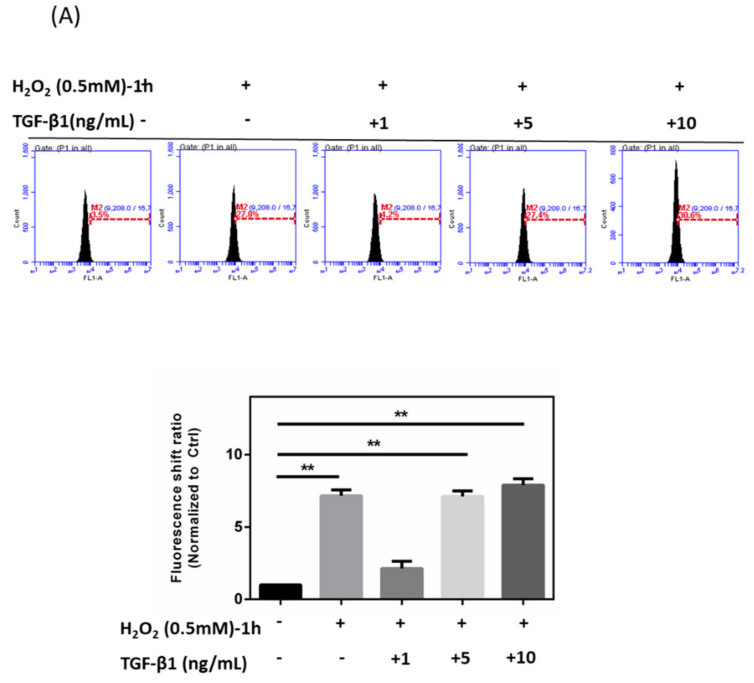

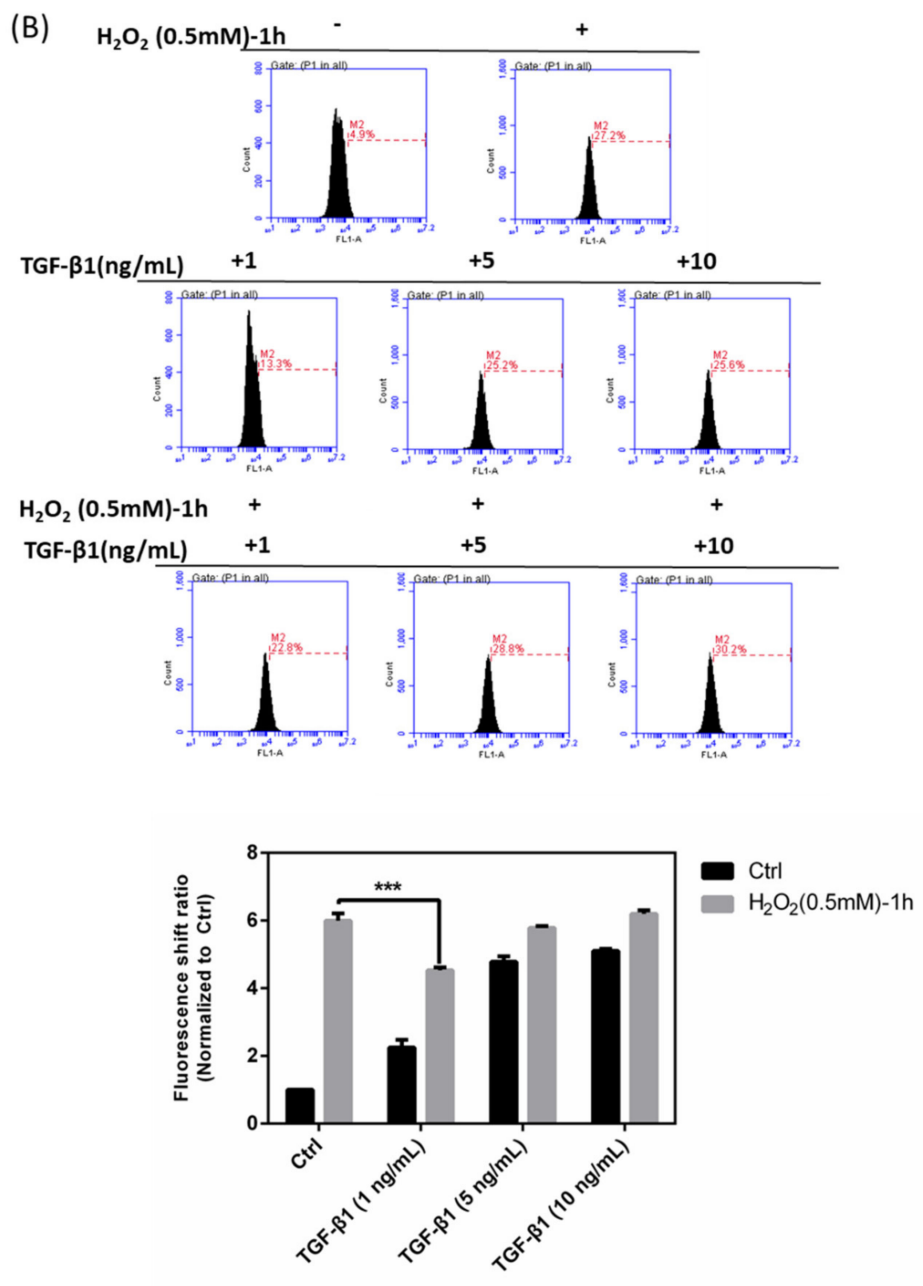

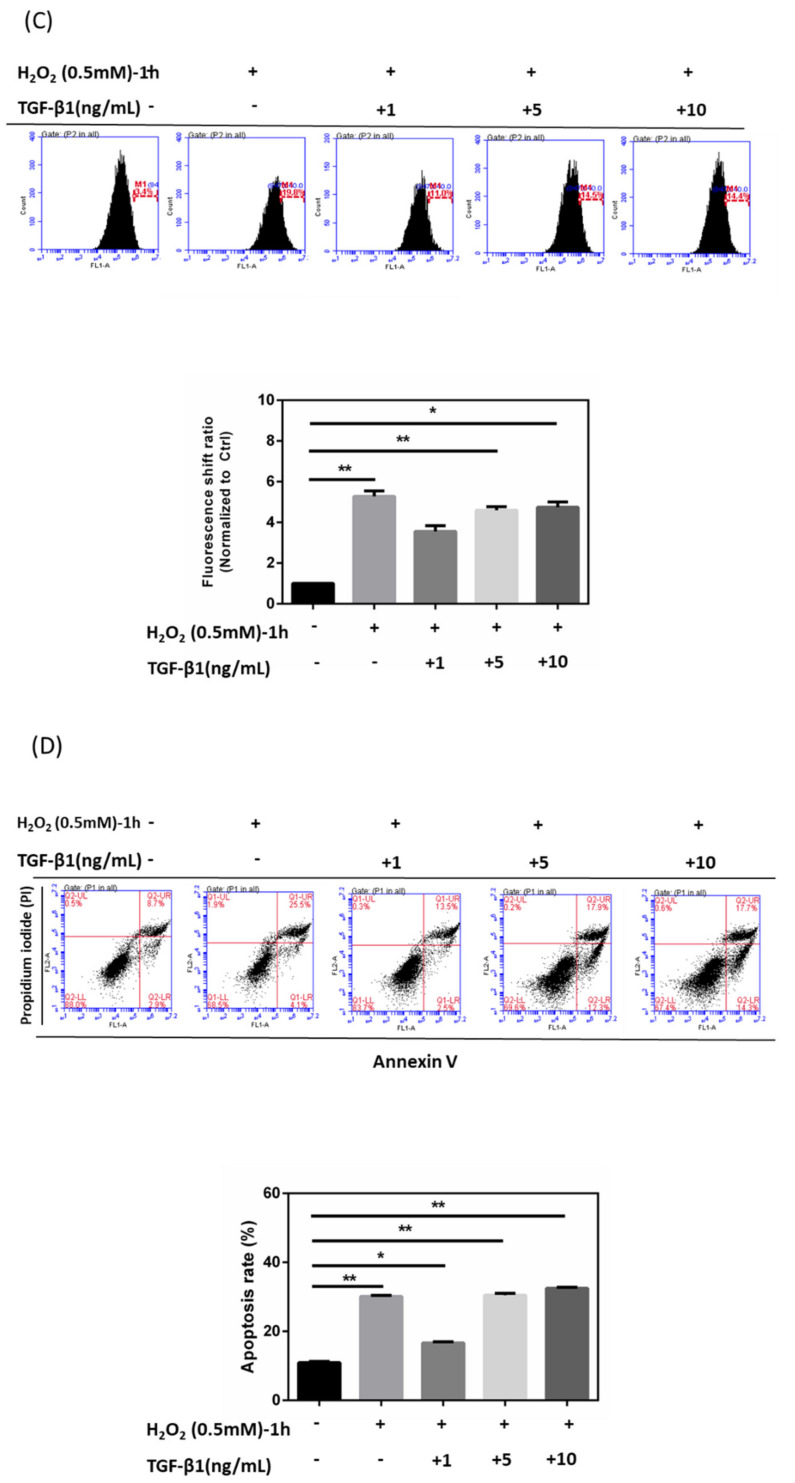
Effects of rhTGF-β1 protein during oxidative stress response of HTMCs. (**A**) Levels of ROS intensity in HTMCs with rhTGF-β1 pretreatment at different concentrations (1, 5, 10 ng/mL) combined with H_2_O_2_ treatment (0.5 mM) for 1 h. Data on rhTGF-β1 (1 ng/mL) pretreatment with H_2_O_2_ showed lower intracellular ROS and indicated protective effects. Histogram values reflect the mean ± SEM. Values in the plots are normalized against the control group (0 min); ** *p* < 0.01 when compared to the control group (*n* = 3, independent experiments). (**B**) Levels of ROS intensity in HTMCs with rhTGF-β1 treatment at different concentrations (1, 5, 10 ng/mL) followed with or without H_2_O_2_ treatment (0.5 mM) for 1 h. Data with rhTGF-β1 (1, 5, 10 ng/mL) only indicated increased ROS levels; however, data for rhTGF-β1 (1 ng/mL) pretreatment with H_2_O_2_ showed lower intracellular ROS and indicated protective effects. Histogram values reflect the mean ± SEM. Values in the plots are normalized against the control group (0 min); *** *p* < 0.001 when compared to the control group (*n* = 3, independent experiments). (**C**) Histograms showing decreased levels of Ca^2+^ content in rhTGF-β1 (1 ng/mL) combined with H_2_O_2_ (0.5 mM) treatment. Values reflect the mean ± SEM, normalized against the control group (0 min); * *p* < 0.05 and ** *p* < 0.01 when compared to the control group (*n* = 3, independent experiments). (**D**) Apoptosis rate for HTMCs with rhTGF-β1 pretreatment at different concentrations (1, 5, 10 ng/mL) combined with H_2_O_2_ treatment (0.5 mM) for 1 h. Cell death decreased with rhTGF-β1 (1 ng/mL) pretreatment followed by H_2_O_2_-induced oxidative stress. Histogram values reflect the mean ± SEM. Values in the plots are normalized against the control group (0 min); * *p* < 0.05 and ** *p* < 0.01 when compared to the control group (*n* = 3, independent experiments).

HTMCs were pretreated with rhTGF-β1 at different concentrations (1, 5, and 10 ng/mL), and intracellular ROS, Ca^2+^ content, and cell apoptosis status were investigated. Following pretreatment with rhTGF-β1 (1, 5, 10 ng/mL), H_2_O_2_ (0.5 mM)-induced oxidative stress was prolonged for 1 h. Data showed that rhTGF-β1 (1 ng/mL) pretreatment reduced the amount of ROS (decreased the peak-shift ratio from 7.5 to 2) and cell apoptosis rate (29.6% to 16%), while it also enhanced survival; groups with rhTGF-β1 (5 ng/mL) pretreatment showed no difference from the H_2_O_2_-only group no matter the fluorescence shift ratio of ROS increase (remained around 7.5 times normalized to Ctrl) or cell apoptosis percentages (29.6% to 30.2%), and groups with rhTGF-β1 (10 ng/mL) pretreatment experienced an increased cell apoptosis rate (29.6% to 32%) and amount of ROS (up to 8.3-fold) (Figure 4A,B,D). Meanwhile, it was shown that groups with rhTGF-β1 (1 ng/mL) experienced a decrease in peak-shift percentages of intracellular Ca^2+^ concentration from 5.5-fold to 3.8-fold. (Figure 4C). Interestingly, although the data surrounding ROS increase with rhTGF-β1 (1, 5, 10 ng/mL)-only groups indicated formation of intracellular ROS, the concentration of 1 ng/mL totally demonstrated its protective effects on HTMCs with H_2_O_2_-induced oxidative stress.

### 3.5. Immunoblot Analysis and Identification of rhTGF-β1 Protein in Oxidative Stress Pathways

**Figure 5:**
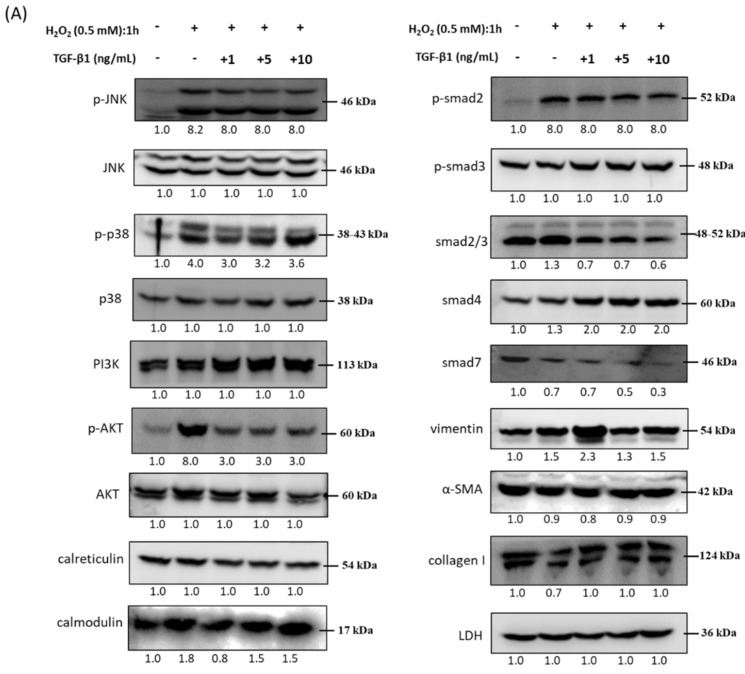

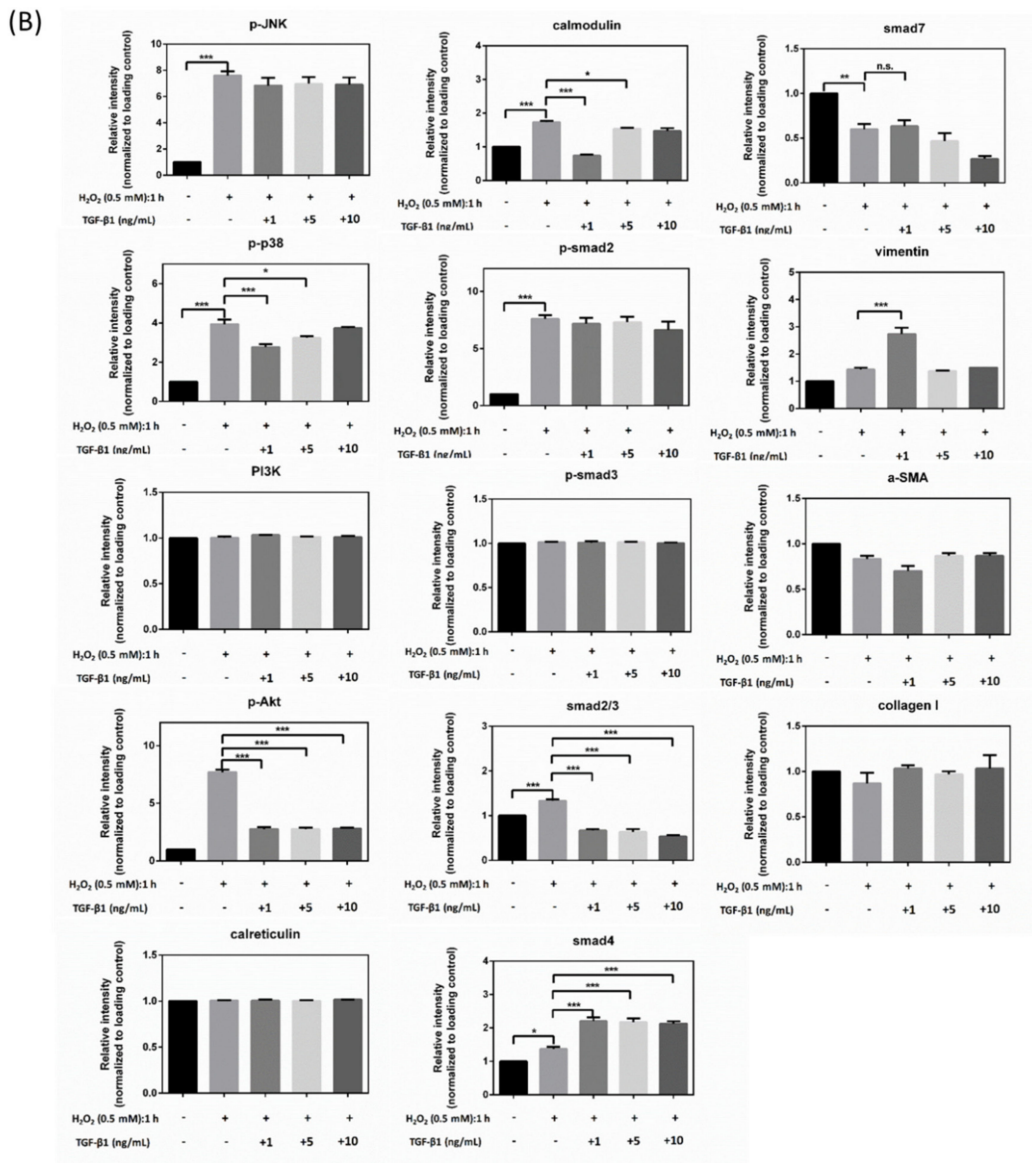
Immunoblot analysis and identification of rhTGF-β1 protein in oxidative stress pathways. (**A**) Cells were collected after treatment with specific rhTGF-β1 concentrations (1, 5, 10 ng/mL) followed by H_2_O_2_ (0.5 mM) treatment for 1 h, and cellular extracts were immunoblotted with p-JNK, JNK, p-p38, p38, PI3K, p-AKT, AKT, calreticulin, calmodulin, p-SMAD2, p-SMAD3, SMAD2/3, SMAD4, SMAD7, vimentin, alpha-smooth muscle actin (α-SMA), and collagen I as shown with the protein bands. Oxidative stress-dependent increases in ECM proteins (vimentin), stress response, and Ca^2+^-modulated proteins were observed over time. Numbers under each protein band reflect the densitometry value. (**B**) The protein expression intensities were quantified in relation to the loading control. Statistic chart were represented as means ± SEM. *, *p* < 0.05; **, *p* < 0.01; ***, *p* < 0.001; n.s., non-significant; (*n* = 3).

HTMCs were pretreated with rhTGF-β1 at different concentrations (1, 5, 10 ng/mL) and further exposed to H_2_O_2_ (0.5 mM) for 1 h. The protein phosphorylation of p38 and AKT decreased with increasing concentrations of rhTGF-β1. It is worth mentioning that the protein level of vimentin was upregulated in the group with rhTGF-β1 (1 ng/mL); this may show not only ECM status but also the structural maintenance taking place in the eyeball. For most of the oxidative stress-response molecules, such as Ca^2+^-related regulators and TGF-β-related SMAD family members, protein level alteration did not show a significant difference among varying concentrations of rhTGF-β1 combined with H_2_O_2_ treatment (Figure 5).

### 3.6. Immunofluorescence Analysis of rhTGF-β1 Protein-Treated HTMCs with or without H_2_O_2_ Exposure (0.5 mM for 1 h)

**Figure 6:**
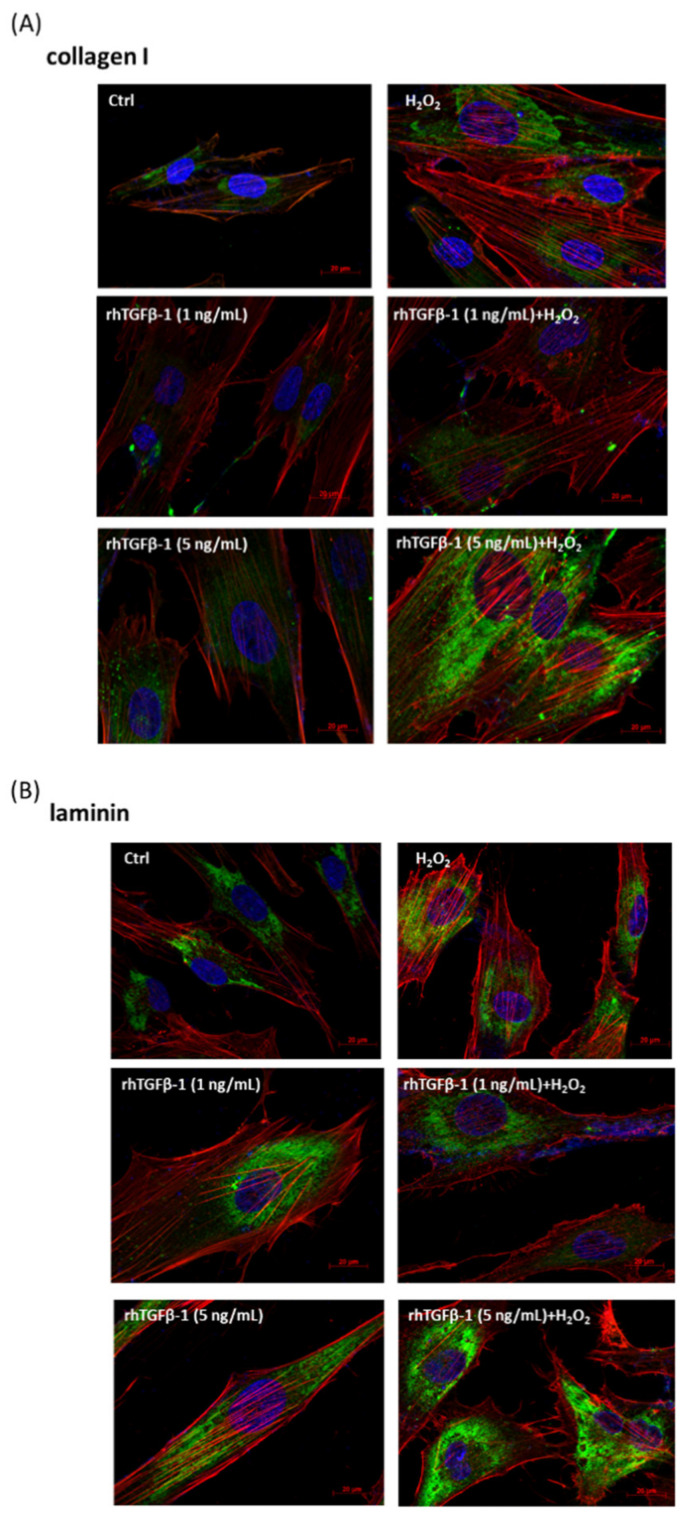
Immunofluorescence analysis of rhTGF-β1 protein-treated HTMCs with or without H_2_O_2_ exposure (0.5 mM for 1 h). Fluorescence photomicrograph of HTMCs with rhTGF-β1 treatment at different concentrations (1, 5 ng/mL) with or without H_2_O_2_ treatment (0.5 mM) for 1 h. HTMCs were treated with specific conditions on 12 mm coverslips fixed and stained with (**A**) collagen I (green), (**B**) laminin (green), phalloidin (red), and DAPI (blue). The same exposure was used for each set of fields, and the images are indicative of three different fields (scale bar = 20 μm).

Subsequently, to further elucidate the role and threshold of TGF-β and whether TGF-β promotes anti-oxidative pathways or triggers much more oxidative stress, thereby leading to severe fibrosis, we established immunofluorescence analysis experiments with rhTGF-β1 protein (1, 5, 10 ng/mL) and rhTGF-β1 protein followed by H_2_O_2_ treatment to track ECM protein markers, specifically collagen I (Figure 6A) and laminin (Figure 6B). Our results suggested that the groups with rhTGF-β1 (1 ng/mL) combined with H_2_O_2_-induced oxidative stress exhibited a lower intensity of collagen I and laminin, implying less progression of fibrosis.

Overall, these results indicate that low concentrations of rhTGF-β (1 ng/mL) act as a cell antioxidant that could shield HTMCs against H_2_O_2_-induced oxidative damage and decrease ECM marker amount.

### 3.7. Effects of shTGF-β1/2 Knockdown in HTMCs with H_2_O_2_ Exposure

**Figure 7:**
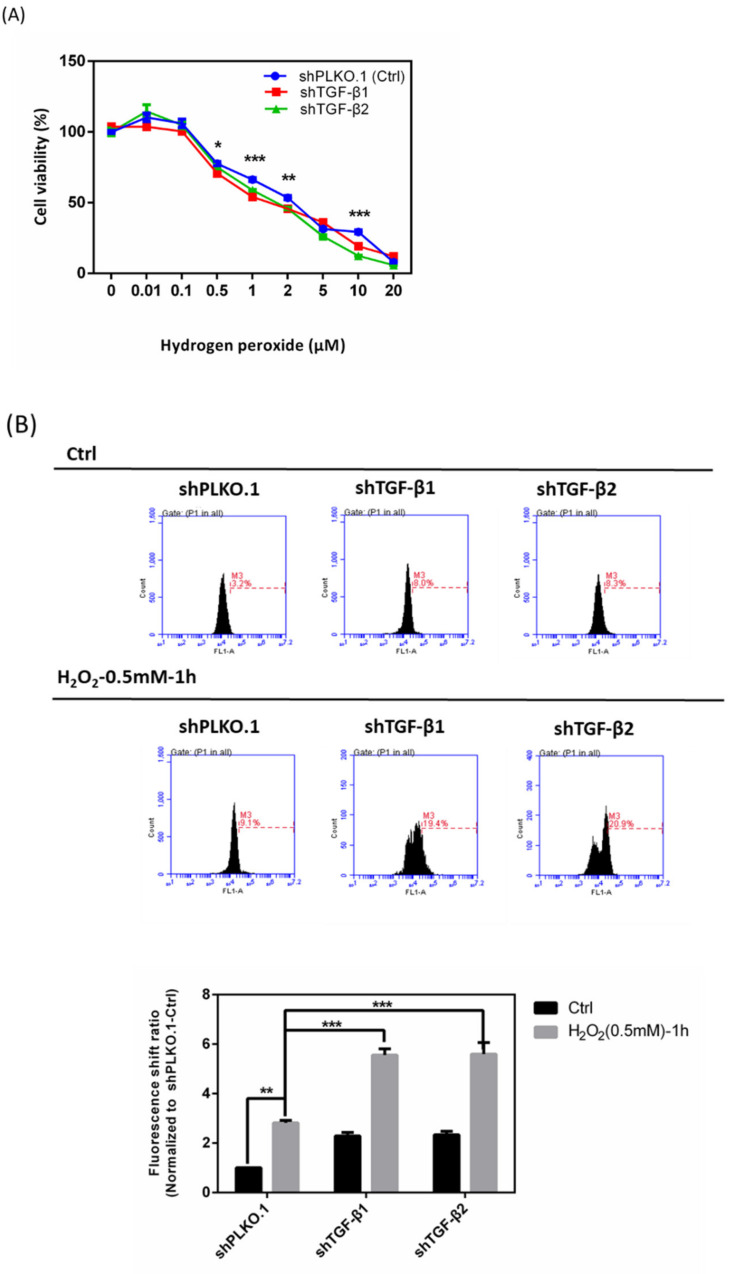

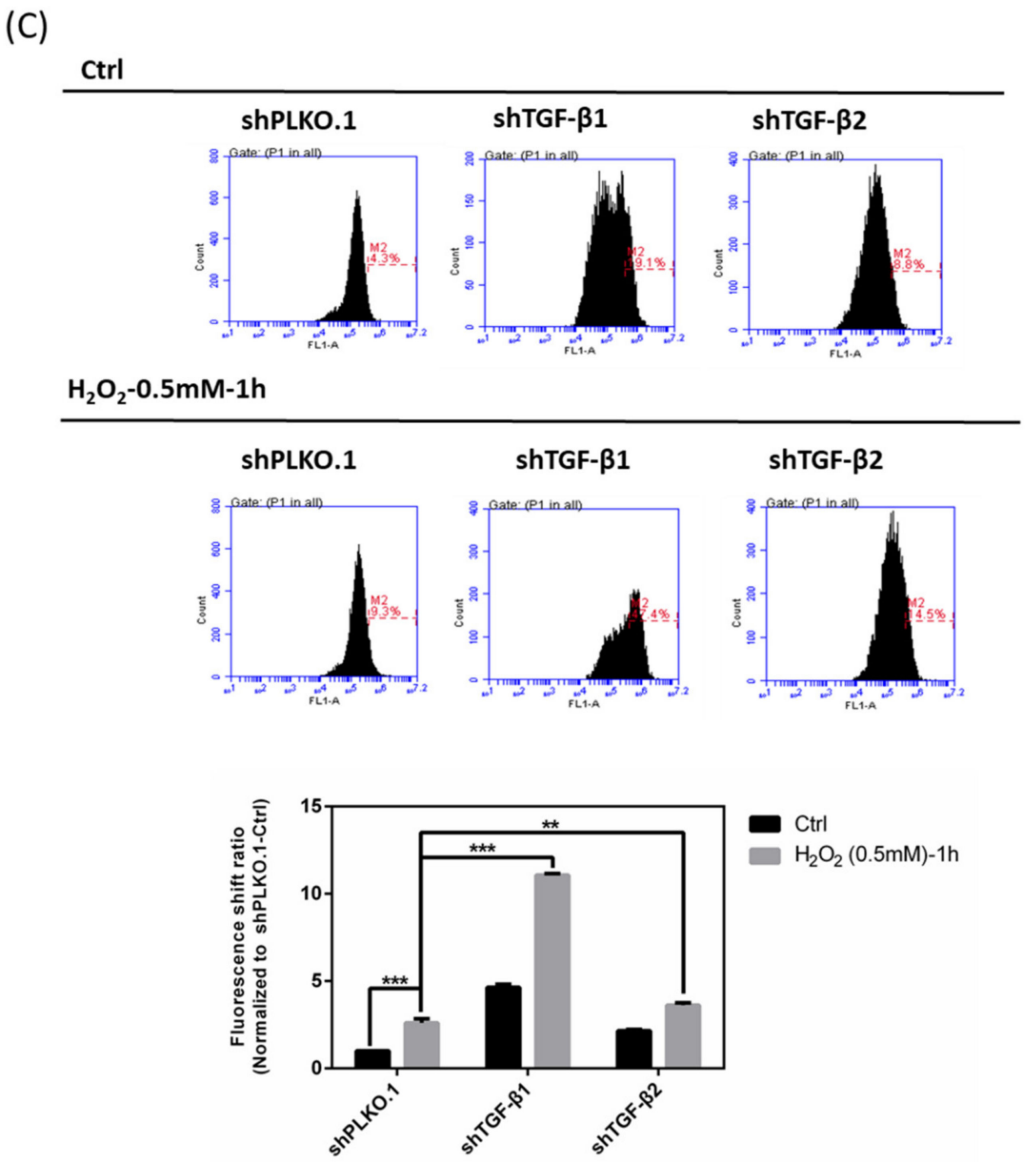

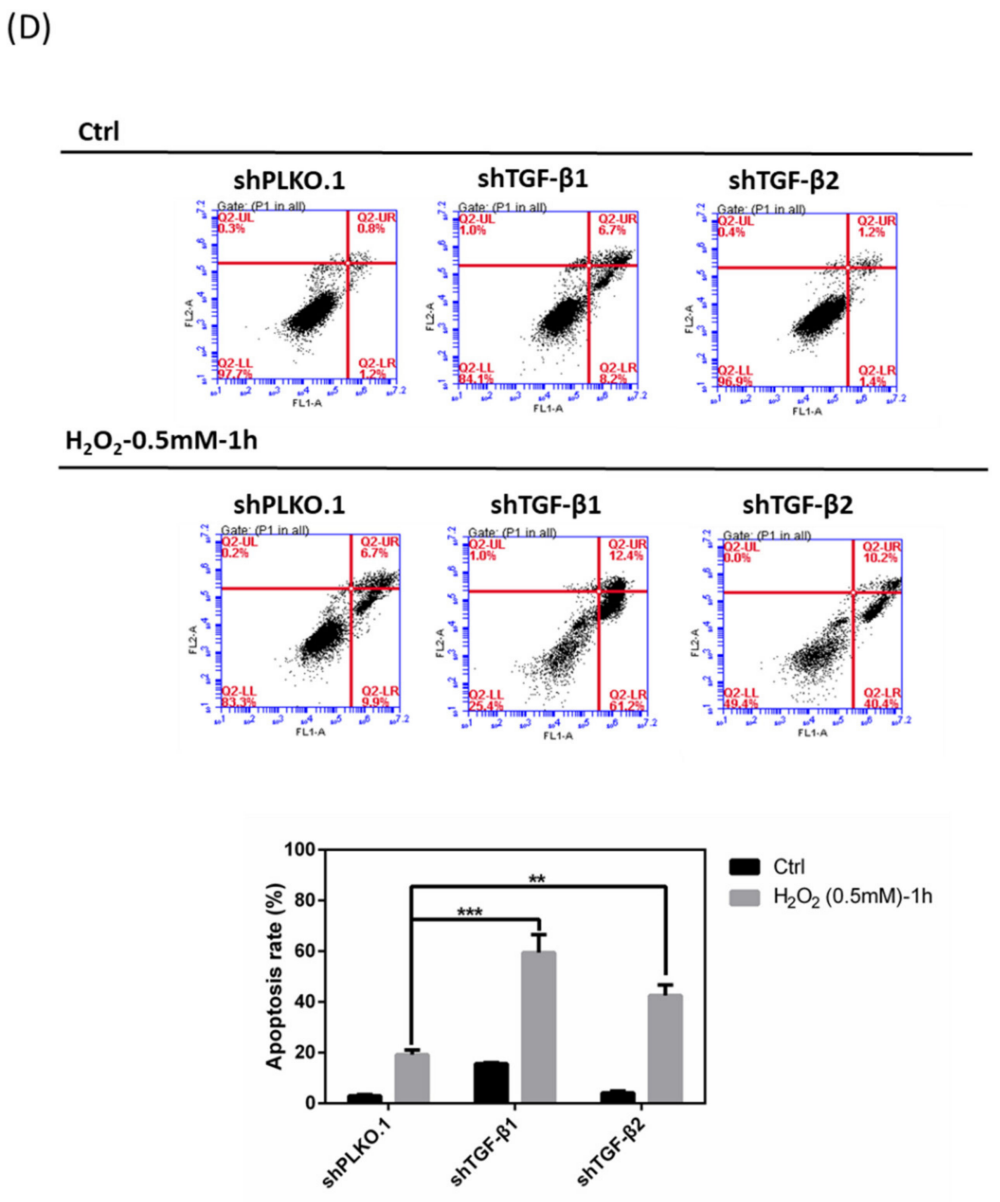
Effects of shTGF-β1 and shTGF-β2 knockdown in HTMCs with H_2_O_2_ exposure. (**A**) TGF-β1/2 knockdown HTMCs had decreased cell viability with H_2_O_2_ at different concentrations for 1 h when compared to the shPLKO.1 group (control). Data reflected that shTGF-β1/2 knockdown HTMCs were more susceptible and sensitive to oxidative stress. Values in the plots are normalized against the control group (shPLKO.1-Ctrl); Statistic chart were represented as means ± SEM. *, *p* < 0.05; **, *p* < 0.01; ***, *p* < 0.001; (*n* = 3, independent experiments). (**B**) Levels of ROS intensity for shTGF-β1/2 knockdown HTMCs with or without H_2_O_2_ treatment (0.5 mM) for 1 h. Data for shTGF-β1/2, especially shTGF-β1, knockdown HTMCs with H_2_O_2_ (0.5 mM) for 1 h showed greater ROS increase. Values in the plots are normalized against the control group (shPLKO.1-Ctrl); Statistic chart were represented as means ± SEM. **, *p* < 0.01; ***, *p* < 0.001; (*n* = 3, independent experiments). (**C**) Histograms depicting increased levels of Ca^2+^ contents in shTGF-β1 and shTGF-β2 knockdown HTMCs combined with H_2_O_2_ (0.5 mM) for 1 h of treatment. Values in the plots are normalized against the control group (shPLKO.1-Ctrl); Statistic chart were represented as means ± SEM. **, *p* < 0.01; ***, *p* < 0.001; (*n* = 3, independent experiments). (**D**) Apoptosis rate for shTGF-β1 and shTGF-β2 knockdown HTMCs combined with H_2_O_2_ treatment (0.5 mM) for 1 h. Cell death increased in shTGF-β1 and shTGF-β2 knockdown groups followed by H_2_O_2_-induced oxidative stress. Values in the plots are normalized against the control group (shPLKO.1-Ctrl); Statistic chart were represented as means ± SEM. **, *p* < 0.01; ***, *p* < 0.001; (*n* = 3, independent experiments).

According to our previously described experimental data, we could now conclude that HTMCs with a low concentration of rhTGF-β1 (1 ng/mL) exhibited protective effects in terms of antioxidation, while elevated concentrations of rhTGF-β1 (5 and 10 ng/mL) showed no benefit and even demonstrated aggravated damage. We then examined whether loss of TGF-β1/2 affects cell viability, intracellular ROS, and Ca^2+^ levels with H_2_O_2_ damage. Cell viability with respect to tolerance of H_2_O_2_ revealed that the shTGF-β1/2 knockdown group was more sensitive and susceptible to H_2_O_2_-induced oxidative stress (Figure 7A). Intracellular ROS significantly increased in the shTGF-β1/2 group with the same oxidative stress induced by H_2_O_2_ as that of shPLKO.1 (control group) (Figure 7B). Furthermore, [Ca^2+^]i was significantly elevated in the absence of TGF-β1/2, especially in the shTGF-β1 group (Figure 7C). Collectively, consistent with the aforementioned results, an increased cell apoptosis rate in the shTGF-β1 and shTGF-β2 groups with H_2_O_2_ treatment was observed (Figure 7D).

### 3.8. Immunoblot Analysis and Identification of shTGF-β1/2 Knockdown HTMCs in Oxidative Stress Pathways

**Figure 8:**
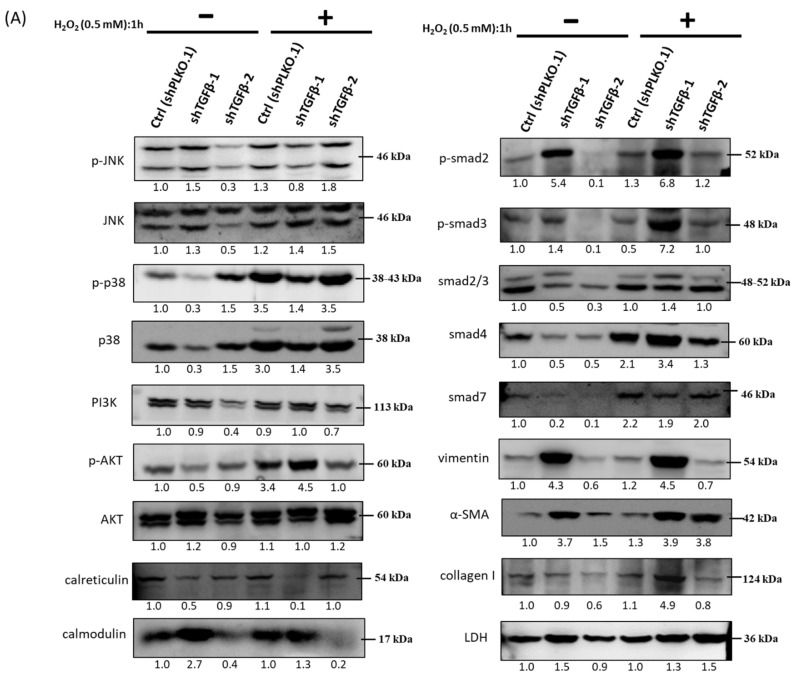

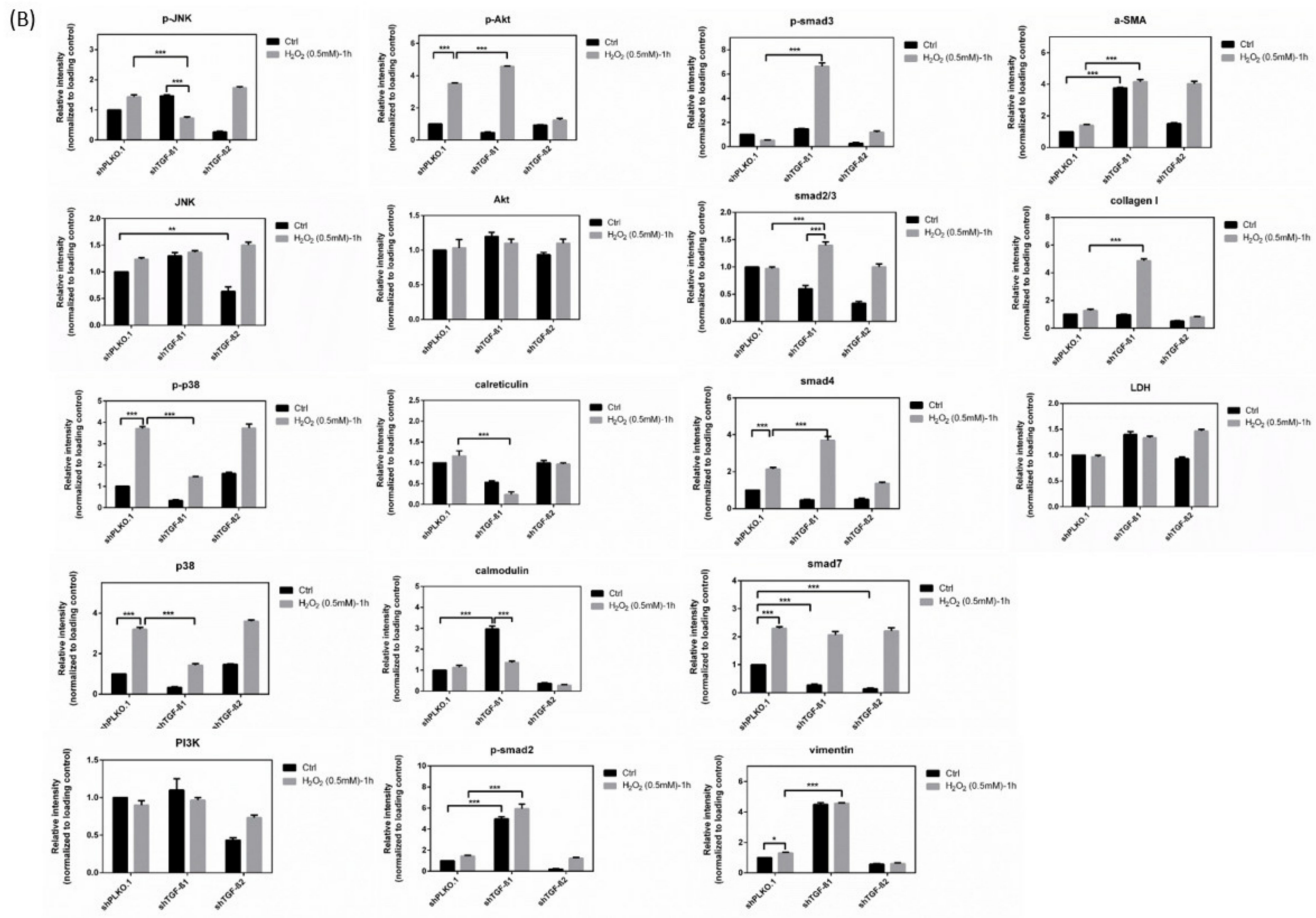
Immunoblot analysis and identification of shTGF-β1/2 knockdown HTMCs in oxidative stress pathways. (**A**) shPLKO.1 (control), shTGF-β1, and shTGF-β2 knockdown HTMCs were collected after treatment with H_2_O_2_ (0.5 mM) induced for 1 h, and cellular extracts were immunoblotted with p-JNK, JNK, p-p38, p38, PI3K, p-AKT, AKT, calreticulin, calmodulin, p-SMAD2, p-SMAD3, SMAD2/3, SMAD4, SMAD7, vimentin, α-SMA, and collagen I, as shown via protein bands. An oxidative stress-dependent increase in ECM proteins, vimentin, and stress response (especially the p-p38 pathway) is evident. Numbers under each protein band reflect the densitometry value. (**B**) The protein expression intensities were quantified in relation to the loading control. Statistic chart were represented as means ± SEM. *, *p* < 0.05; **, *p* < 0.01; ***, *p* < 0.001 (*n* = 3).

As in a previous report, we found a reduction in Nrf2, ALDH3A1, HO-1, and lamin B1, while the promotion of Keap1 and HIF-1α in shTGF-β1/2 knockdown RGCs was associated with neuroprotection pathways. Protein level alteration assays showed that shTGF-β1/2 knockdown HTMCs resulted in higher levels of p-p38, collagen I, and α-SMA, which could be linked to a progressive fibrosis situation (Figure 8). The level of other related proteins, such as those from the TGF-β-related SMAD family, decreased due to knockdown of TGF-β1 and TGF-β2, and cell signal transduction was inhibited.

### 3.9. Effects of sip38 MAPK Knockdown in HTMCs with H_2_O_2_ Exposure

**Figure 9:**
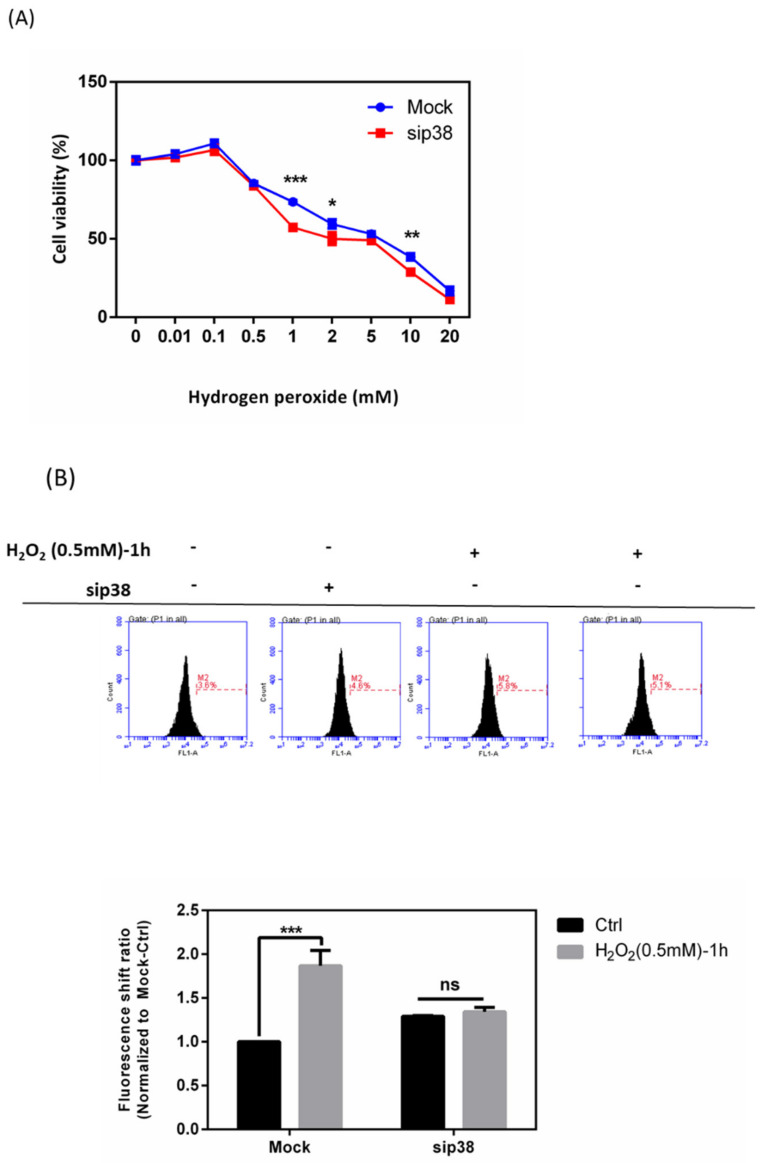
Effects on sip38 MAPK knockdown HTMCs through H_2_O_2_ exposure. (**A**) sip38 MAPK knockdown HTMCs decreased cell viability with H_2_O_2_ at different concentrations for 1 h when compared to the si-control (mock) group. Data reflect that ssip38 MAPK knockdown HTMCs were more susceptible and sensitive to oxidative stress. Values in the plots are normalized against the control group (Mock-Ctrl); Statistic chart were represented as means ± SEM. *, *p* < 0.05; **, *p* < 0.01; ***, *p* < 0.001; (*n* = 3, independent experiments). (**B**) Levels of ROS intensity in sip38 MAPK knockdown HTMCs with or without H_2_O_2_ treatment (0.5 mM) for 1 h. Data show that insip38 MAPK knockdown HTMCs had increased ROS levels; H_2_O_2_ (0.5 mM) treatment for 1 h led to no significance increase. Values in the plots are normalized against the control group (Mock-Ctrl); Statistic chart were represented as means ± SEM. ***, *p* < 0.001; ns, non-significant; (*n* = 3, independent experiments).

The data in Figure 2 show that p-p38 was significantly increased after treatment with 0.5 mm H_2_O_2_. We then attempted to investigate the significance of p38 MAPK for the antioxidative potential of HTMCs transiently transfected with siCtrl or p38 MAPK siRNA (sip38 MAPK). Transfection of sip38 MAPK resulted in more than a 70% reduction in p38 MAPK activation in HTMCs when compared to siCtrl-transfected HTMCs. As shown in Figure 9A, sip38 MAPK-transfected HTMCs were significantly decreased in terms of cell viability when exposed to H_2_O_2_-related oxidative stress. In addition, the ROS level in the sip38 MAPK group with H_2_O_2_ treatment showed no significant increase (Figure 9B). These data implied that the modulation of the p38 MAPK pathway may be directly mediated by the irregular increase in ROS and modulate other downstream signal transduction pathways.

### 3.10. Immunoblot Analysis and Identification of sip38 MAPK Knockdown HTMCs in Oxidative Stress Pathways

**Figure 10:**
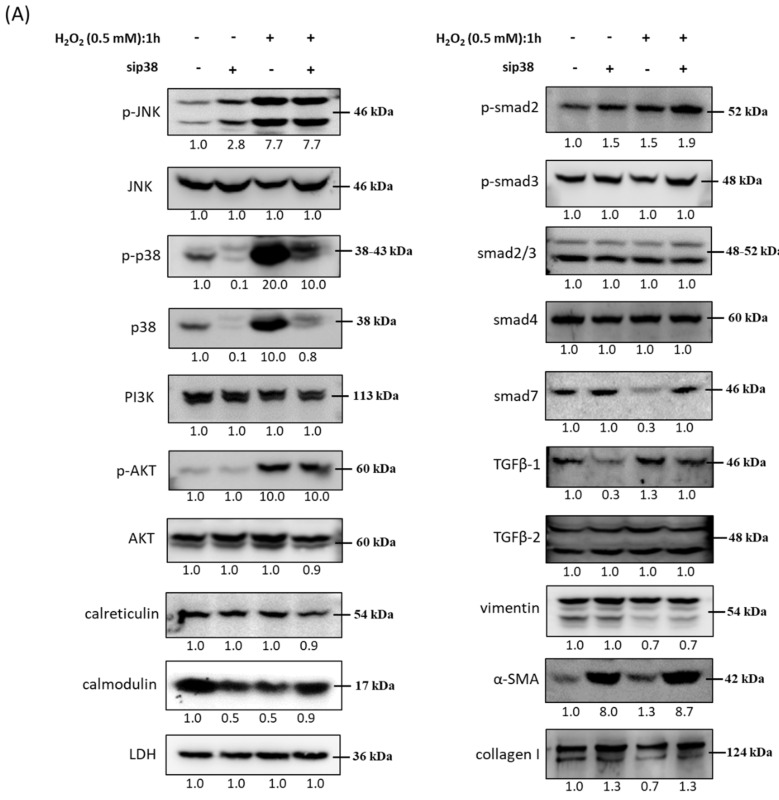

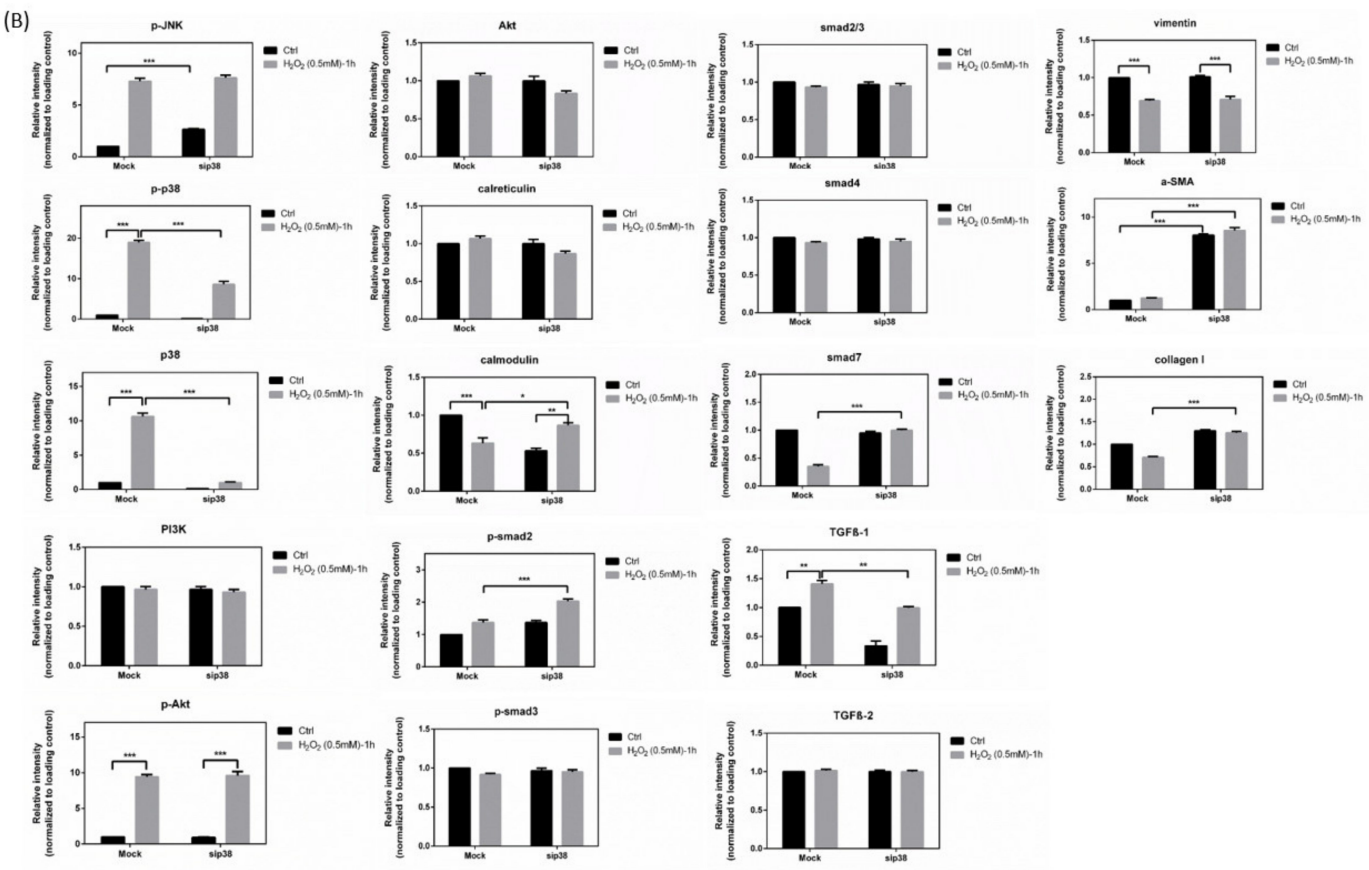
Immunoblot analysis and identification of sip38 MAPK knockdown HTMCs in oxidative stress pathways. (**A**) Si-control (mock) and sip38 MAPK knockdown cells were collected after treatment with H_2_O_2_ (0.5 mM) for 1 h, and cellular extracts were immunoblotted with p-JNK, JNK, p-p38, p38, PI3K, p-AKT, AKT, calreticulin, calmodulin, p-SMAD2, p-SMAD3, SMAD2/3, SMAD4, SMAD7, TGFβ-1, TGFβ-2, vimentin, α-SMA, and collagen I, as shown via protein bands. There was an oxidative stress-dependent increase in ECM proteins (α-SMA) and stress response, while TGF-β family members were observed to decrease. Numbers under each protein band reflect the densitometry value. (**B**) The protein expression intensities were quantified in relation to the loading control. Statistic chart were represented as means ± SEM. *, *p* < 0.05; **, *p* < 0.01; ***, *p* < 0.001 (*n* = 3).

A previous study [26] indicated that the development of type 1 collagen triggered by TGF-β2 is suppressed by inhibition of p38 and followed by partial inactivation of SMAD2/3 in HTMCs. To further examine the impact of p38 MAPK on other related protein markers during oxidative stress of HTMCs, immunoblot analysis was performed to identify the mechanisms. In Figure 10, the protein level of TGF-β1, but not TGF-β2, was diminished in the sip38 MAPK knockdown group when compared to the si-control group. Notably, the protein level of α-SMA was significantly increased in the sip38 MAPK group and further elevated in the sip38 MAPK with H_2_O_2_ treatment. Altogether, these results suggested that the p38 MAPK pathway is associated with TGF-β1 signal transduction, and oxidative stress results in TGF-β overproduction, leading to HTMC fibrosis by activating p38 MAPK-mediated ECM protein.

### 3.11. A Hypothetical Model Detailing the Role of the TGF-β-Related Oxidative Stress Pathway in HTMCs

**Figure 11:**
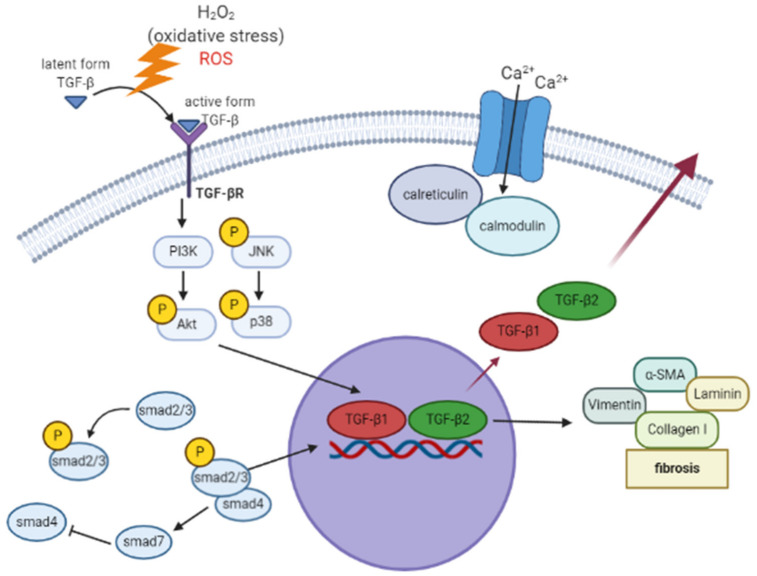
TGF-β-related oxidative stress signaling pathway in HTMCs (created in BioRender.com).

In the H_2_O_2_-mediated oxidative state, the increased ROS activates TGF-β conversion from the latent form to the active form to induce downstream signaling. Upregulated TGF-β in HTMCs subsequently induces phosphorylation of JNK, p38, and AKT, further inducing the increase in TGF-β1 and TGF-β2 level, which causes the appearance of downstream fibrosis markers (vimentin, α-SMA, collagen I, laminin). Increased fibrosis markers may damage the HTMC drainage system and enhance resistance to aqueous humor cycling, resulting in glaucoma, optic nerve death, and visual loss (Figure 11).

## 4. Discussion

In our previous study, an in vitro study of RGCs, we showed the role of TGF-β1 in oxidative stress-induced RGCs. Through promoting cell antioxidant and neuroprotection pathways, including Nrf2/-1 alpha/ALDH3A1/HO-1 signaling, TGF-β1 protected RGCs from H_2_O_2_-induced oxidative stress [36]. Thus, on the basis of these results, to better understand the role in and effects of TGF-β on ocular tissues, we further focused on the trabecular meshwork, and we investigated the molecular mechanism responsible for oxidative stress via a series of cell functional assays and protein level alterations. TGF-β has either exceptional cytoprotective qualities or acts as a damage promoter, and it modulates many cell signaling pathways [16,19,22,37,38,39,40,41,42,43,44,45]. Herein, we explored the role of TGF-β in HTMCs, specifically how its levels affect cell biology in HTMCs during oxidative stress.

We developed a series of experiments to elucidate the function of TGF-β to better understand whether the loss of TGF-β with induced oxidative stress has a direct impact on the development of oxidative pathobiology. Our study revealed that loss of TGF-β in HTMCs following H_2_O_2_ treatment has several negative impacts: (a) it has a major effect on cell viability; (b) it permits increase of ROS; (c) it increases intracellular Ca^2+^ influx; (d) it accelerates the process of apoptosis; (e) it overstimulates ECM proteins (Figure 7 and Figure 8). Externally added oxidative stressors, such as H_2_O_2_, facilitate these phenomena, and, surprisingly and interestingly, adding the correct concentration of TGF-β (1 ng/mL) to HTMCs reverses these processes. We propose that the loss of TGF-β, especially TGF-β1, may be associated with HTMC pathobiology resulting from H_2_O_2_-induced oxidative stress. In studying how and to what extent the relative upregulation of TGF-β during oxidative stress-induced HTMCs influences etiopathological alteration, we observed that intracellular ROS and [Ca^2+^] increased with H_2_O_2_ exposure duration (Figure 1). Oxidative stress typically results from excess ROS production due to defective antioxidants under normal physiological conditions, which in turn destroys proteins, lipids, mitochondria, and DNA, leading to further impairment of cellular integrity and functionality. Subsequent to high IOP, through acute or chronic conditions, oxidative stress can be produced [46]. Elevated IOP, as a function of HTMC abnormalities, leads to an increase in resistance to aqueous humor outflow. HTMCs are extremely susceptible to oxidative damage [8]. Previous research suggests that the loss of physiological functions is due to oxidative stress. HTMCs are in directly contact with H_2_O_2_ and TGF-β in aqueous humor. TGF-βs have been measured at high levels in the aqueous humor of glaucomatous eyes [13,15,16,47]. Our study shows that TGF-β activity is increased with H_2_O_2_-oxidative stress treatment (Figure 2), which leads to stress fiber formation (Figure 3) and is associated with a reduced level of p38 MAPK (Figure 9 and Figure 10), which implies that p38 protein modulates the downstream TGF-β pathway. ROS are TGF-β activators; we assume that secreted TGF-β is activated in aqueous humor by existing ROS. In addition, activated TGFβs induce oxidative stress through NOX4 activation, and higher ROS and TGF-β act negatively on the biology of HTMCs in turn.

The absence or over-presence of the complicated protein, TGF-β, accompanied by oxidative stress can lead to an enhanced amount of ROS and cell abnormalities. We also observed that different concentrations of added TGF-β1 cause totally different and bilateral effects: (a) pretreatment with rhTGF-β1 (1 ng/mL) followed by H_2_O_2_ treatment reduced amount of ROS, the cell apoptosis rate, and the protein expression ofcollagen I and laminin, while enhancing survival; (b) groups with rhTGF-β1 (5 ng/mL) pretreatment showed no difference to the H_2_O_2_-only group regardless of ROS increase or cell apoptosis percentages, while there was elevated intensity of collagen I and laminin; (c) groups with rhTGF-β1 (10 ng/mL) pretreatment experienced an increased cell apoptosis rate and amount of ROS (Figure 4, Figure 5 and Figure 6).

The p38 MAPK signaling pathway might play a role in the adverse effects of ROS. Numerous studies have shown that p38 MAPK activation plays a significant role in multiple stimulus-induced apoptosis [26,48]. Miyuki Inoue-Mochita et al. indicated that p38 inhibition caused TGF-β2–induced type 1 collagen downregulation. This implies that the regulation of p38 and TGF-β leads to synchronous alterations and may be implicated in the cell cycle pathway, which acts as positive feedback of the signaling transduction. Furthermore, our data demonstrated a decrease in TGF-β1 in the sip38 group and a decrease in p-p38/p38 in the shTGF-β1 group. Meanwhile, sip38 combined with H_2_O_2_ damage resulted in increased cell survival and intracellular ROS that was downregulated when compared to the si-control group. A broad family of proteins triggered by a wide variety of proinflammatory cytokines and environmental stressors are included in MAPKs. In various cellular processes, such as proliferation, apoptosis, gene regulation, differentiation, and motility, MAPKs play pivotal roles. Recent studies have focused on the p38 MAPK pathway in oxidative stress [49].

As mentioned before, TGF-β1 protected RGCs from H_2_O_2_-induced oxidative stress. It is noteworthy that the data showed that 5 ng/mL TGF-β1 appeared to be the optimal concentration of RGC exposure to oxidative stress. Nevertheless, the conditions in HTMCs showed that the concentration of 5 ng/mL became negative and harmful. These results imply that HTMCs are more susceptible to oxidative stress than RGCs, which is consistent with the results [36]. Our findings showed that the order of protein level alterations in various ocular regions (i.e., cornea, conjunctiva, uvea, sclera, and retina) was according to the ratio of protein level alterations during ischemia–reperfusion injury, and the retina exhibited tolerance that is comparable to that of other tissues [50].

Collectively, our study reinforces the potential role of TGF-β in the oxidative stress pathway. In order to prevent adverse pathological processes and preserve HTMC integrity and function, the low TGF-β concentrations may be significant. However, further detailed studies are necessary to reveal the role of TGF-β1 and TGF-β2, as well as up/downstream signal transduction. However, on the basis of our experimental data, we suggest that the control of TGF-β as a therapeutic intervention to blunt HTMC pathobiology and/or avoid the oxidative stress process may be a possible cause of the etiology and progression of HTMC pathobiology, probably improving increased IOP-mediated glaucoma.

## Data Availability

Data sharing not applicable.
